# Evidence gap map of performance measurement and management in primary care delivery systems in low- and middle-income countries – Study protocol

**DOI:** 10.12688/gatesopenres.12826.2

**Published:** 2018-11-02

**Authors:** Wolfgang Munar, Birte Snilstveit, Jennifer Stevenson, Nilakshi Biswas, John Eyers, Gisela Butera, Theresa Baffour, Ligia E. Aranda

**Affiliations:** 1 Milken Institute School of Public Health, Department of Global Health, George Washington University, Washington, DC, 20052, USA; 2 International Initiative for Impact Evaluation (3Ie), London International Development Centre, London, WC1H 0PD, UK

**Keywords:** Accountability, Evidence gap maps, Implementation strategies, Low- and middle-income countries, Performance measurement and management systems, Primary Care delivery systems, Quality of care, Safety

## Abstract

**Background** **. **For the last two decades there has been growing interest in governmental and global health stakeholders about the role that performance measurement and management systems can play for the production of high-quality and safely delivered primary care services. Despite recognition and interest, the gaps in evidence in this field of research and practice in low- and middle-income countries remain poorly characterized. This study will develop an evidence gap map in the area of performance management in primary care delivery systems in low- and middle-income countries.

**Methods.** The evidence gap map will follow the methodology developed by 3Ie, the International Initiative for Impact Evaluation, to systematically map evidence and research gaps. The process starts with the development of the scope by creating an evidence-informed framework that helps identify the interventions and outcomes of relevance as well as help define inclusion and exclusion criteria. A search strategy is then developed to guide the systematic search of the literature, covering the following databases: Medline (Ovid), Embase (Ovid), CAB Global Health (Ovid), CINAHL (Ebsco), Cochrane Library, Scopus (Elsevier), and Econlit (Ovid). Sources of grey literature are also searched. Studies that meet the inclusion criteria are systematically coded, extracting data on intervention, outcome, measures, context, geography, equity, and study design. Systematic reviews are also critically appraised using an existing standard checklist. Impact evaluations are not appraised but will be coded according to study design. The process of map-building ends with the creation of an evidence gap map graphic that displays the available evidence according to the intervention and outcome framework of interest.

**Discussion** **. **Implications arising from the evidence map will be discussed in a separate paper that will summarize findings and make recommendations for the development of a prioritized research agenda.

## Background

The critical role that primary care delivery systems can play in helping achieve desirable societal goals in low- and middle-income countries (LMIC) has been widely recognized. Given their potential to serve as first points of contact for continuous, coordinated, comprehensive and people-centered health services, high-performing primary care systems are a necessary element for the achievement of the sustainable development goals, the operationalization of calls for universal health coverage, and the management of global pandemics ^1– 3^. While considerable research is available on primary health care and its constitutive elements, it is not clear which approaches are most effective to ensure that primary care systems consistently deliver safe and quality services, that harmful services are not delivered, that primary care delivery systems acquire the capabilities required for continuous improvement, and that all of the above add up to improved population health and other socially valued outcomes.

The objective of this study is to identify and describe existing evidence on the effects of interventions in the area of performance measurement and management in primary care delivery systems in LMICs and, also, to provide easy access to such evidence for relevant decision makers. The resulting evidence gap map (EGM) will inform the development of a prioritized research agenda for primary care delivery systems in LMIC.

### Why is this study relevant to research, policy and practice in LMICs?

There are multiple approaches, frameworks, and conceptualizations for characterizing health systems, measuring and managing their performance, and typifying health system interventions. The study uses a multidisciplinary approach to identify and characterize the relevant literature from different fields and disciplines such as organizational science, development economics, behavioural science, health systems research, and public-sector management.

Organizational performance refers to the results generated by an organization and measured against its intended aims. In private sector organizations, performance can be a function of profits, organizational efficiency and effectiveness, quality of goods and services, market share, and customer satisfaction. In public administration, the definition of organizational performance has evolved with the changing framings for the role of the State in the production and delivery of public goods and services ^4^. Historically, governments initially emphasized aspects of performance such as the control of inputs and the compliance with standards. Subsequent framings shifted, first, towards a focus on the quantity and quality of outputs, productivity, and efficiency and, in recent years, to outcomes and policy impacts and, in the case of the health sector, to social values like patient- or people-centered health services and equity ^4– 7^.

On the research side, the theories of organizational performance have followed, in general, the evolution of the practice of performance management in high-income countries. According to Talbot ^8^, an initial set of theories and frameworks were focused on characterizing associations between individual elements of performance and organizational effectiveness. Afterwards, researchers focused on excellence, quality and organizational culture which led to the development of a first wave of models of performance measurement and management. These models did not account for differences between public and private sector dimensions of performance but were nonetheless adopted by governments around the world. In the 90s, the focus shifted from theoretical perspectives about organizational performance to interest in how to measure goal achievement in public and private sector organizations using performance models such as the Balance Scorecard and others ^9– 11^. Interest in performance measurement and management spread around the world, and international comparisons and benchmarking of performance flourished in various sectors such as governance ^12, 13^, health ^14^, and education ^15^.

During the last 40 years, innovations in performance measurement and management in the health sector have been prevalent in the United Kingdom, Canada, Australia, New Zealand, Sweden and the US, among other countries ^16– 19^. Amplified by multilateral finance organizations and some bilateral agencies, performance measurement and management systems have spread among LMIC, sometimes as central aspects of large-scale public-sector reforms and, also, as stand-alone health sector reforms. Some elements of performance measurement and management have spread more than others particularly performance-based financing, pay-for-performance, performance budgeting and contracting and the use of financial incentives (defined below).

The spread of the practice and research of performance measurement and management has also affected the global health architecture and its governance. The interest among donor governments, multilateral finance institutions, bilateral agencies, and global philanthropies started shifting since the late 90s from a focus on funding inputs towards an interest on the production of measurable results aid effectiveness, and accountability. Such shifts in preferences contributed to the emergence of new global organizational forms and partnerships such as the Global Fund to Fight HIV/AIDS, Tuberculosis and Malaria, GAVI the Vaccine Alliance, the Global Finance Facility, and the Mesoamerican Health Facility, to name a few.

In terms of effectiveness, 40 years of research on performance measurement and management have shown that, despite many challenges, such systems can be effective ^20– 25^. There is evidence, also, of the generation of unintended effects in the public and private sectors ^22, 26– 29^.

In the area of health systems research, research conducted to date by the Cochrane Collaboration has generated approximately 200 systematic reviews addressing the effective organization of health services. While the majority of these have been focused on issues of relevance to high-income countries research and policy, there is a growing portfolio of reviews focused on delivery and financial arrangements, as well as implementation strategies in LMIC ^30– 38^.

## Study objectives

This evidence gap map aims to identify and describe the existing evidence on the effects of interventions in the area of performance measurement and management in primary care delivery systems in LMICs. Also, to identify evidence gaps where new primary studies or systematic reviews could add value and provide easy access to the best available existing evidence on intervention effects in this area. The resulting EGM will inform the development of a prioritized research agenda for primary care delivery systems in LMICs.

## How performance measurement and management may work in primary care delivery systems

**Figure 1.**
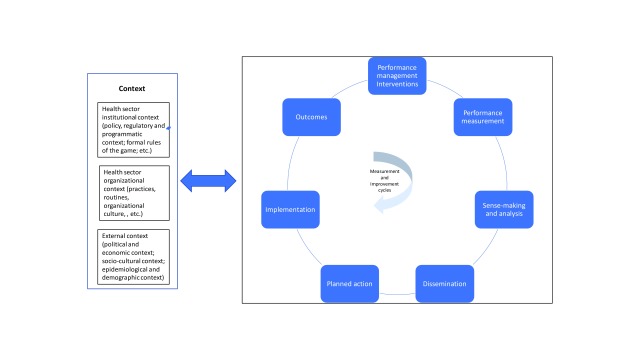
Generic performance measurement and management system.

The components of a generic, performance management system are delineated in Figure 1 and represent an adaptation of two frameworks. The first, is a generic framework of public management in public sector organizations developed by Pollitt ^27^ and the second is the result of ongoing research on primary care performance in Mesoamerica led by one of this study’s authors (WM) ^39^. Framework components include: 1) A context in which various policies, organizations, programs and health interventions coexist with system actors and stakeholders; 2) one or more performance management interventions; 3) activities for measuring the results from the implementation of primary care policies and programs, and its ensuing data; 4) a process through which raw performance data is made sense of and transformed into performance information; 5) dissemination of performance information among system actors and stakeholders with the intent of making it actionable; 6) performance information use, misuse or non-use; 7) implementation of planned action based on the use of performance information; and, 8) the effects from the implementation of planned action and clinical and managerial improvements (proximal processes, outputs and outcomes, and distal, societal and population-level outcomes).

However, the production of actual, measurable performance is a complex, dynamic phenomenon. Real performance can be very hard if at all possible to observe. Its measurement is characterized by lags between the introduction of interventions, the production of effects, and their measurement. These delays can also create a disconnect between action, measurement and results. Once measurement occurs and performance information is available, system actors and stakeholders can respond to the perceived performance gap by using, not using, or misusing such information ^40, 41^. To be effective, performance information needs to apprise subsequent organizational action. Changes in strategic direction or operational tactics would also have to be effectively implemented for outcomes to be generated.

Based on the above theoretical and practice-oriented considerations, the study defines performance measurement and management in a primary care delivery system as the introduction of management systems focused on measuring organizational processes, outputs and outcomes with the proximal aim of informing the introduction of clinical, managerial, programmatic and policy changes and the ultimate goal of contributing to socially valued, population level health outcomes.

**Figure 2.**
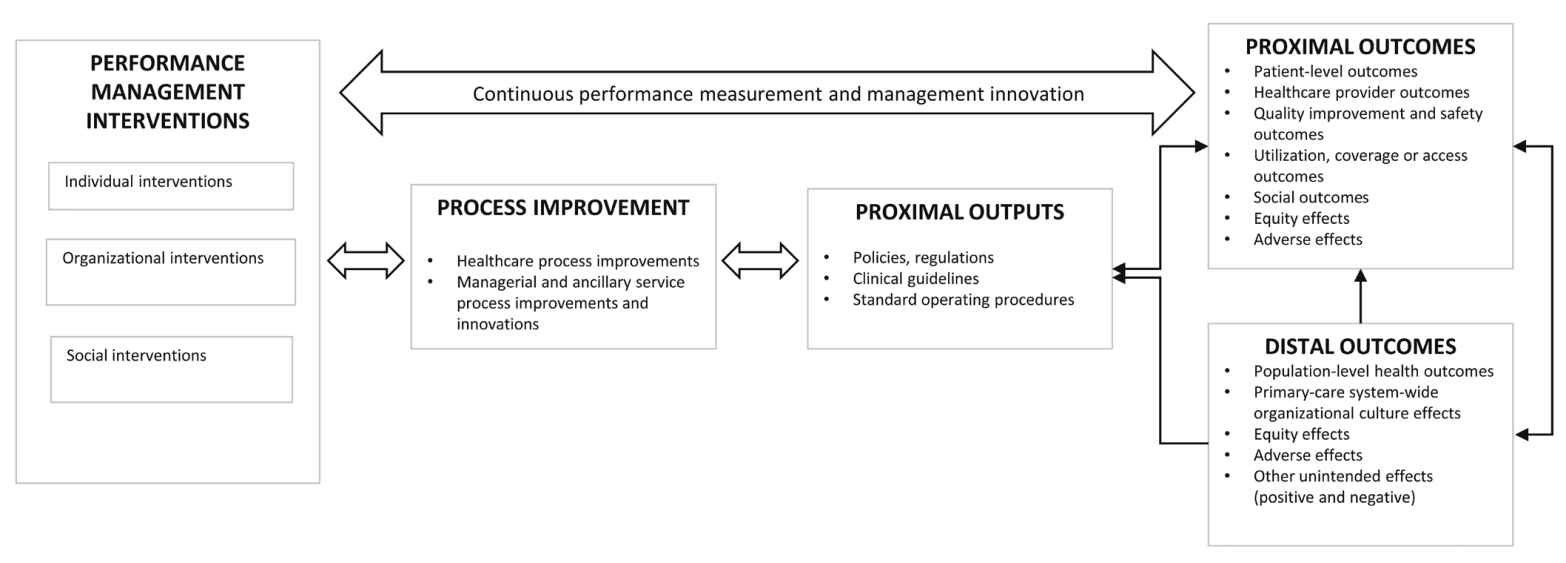
Performance measurement and management framework in primary care delivery systems.

Figure 2 provides a framework which maps out three broad classes of performance measurement and management interventions, the assumed associated process improvements and outputs, and proximal and distal outcomes. The framework highlights some of the dynamic and complex relationships between interventions and outcomes and characterizes a process of multi-level change in a primary care delivery system. The process of change described in this framework adheres to the following logic:

• Performance management interventions operating at individual, organizational, and social levels can initially trigger short-term changes in healthcare as well as in managerial and ancillary service process improvements. Such changes would be the result of short cycles of experimentation with technological, managerial and clinical innovations ^42, 43^.
• The repetition of these cycles through time, and the utilization of the information derived from performance measurement by system actors would lead to the generation of proximal outputs such as policies, regulations, clinical guidelines, and standard operating procedures, as well as negative or unintended outcomes.
• If effectively implemented, these new routines and processes would lead to intended and unintended proximal outcomes including changes in the behaviors of healthcare providers, primary care managers, and policy-makers. Proximal health effects could include the adoption of improved clinical behaviors by providers; quality improvement and safety outcomes at the patient- and facility-levels; increased service utilization and effective coverage; positive and negative equity effects; and, adverse or unintended effects. Examples of negative or unintended effects of performance management interventions have been reported in the literature, including gaming, shirking and cream-skimming ^25, 44– 47^.
• If sustained through time and effectively implemented, additional desirable outcomes from iterative cycles of innovation, measurement and improvement may include increased retention of the workforce; increased productivity and efficiency; or improved equity, among others.
• Continuous cycles of performance measurement and management would also lead to the emergence or reinforcement of organizational-level capabilities and resources that could sustain performance improvements at higher-levels within the primary care system leading to, in some but not all instances, reinforcing cycles of improvement and organizational learning.
• The reiteration of these reinforcing cycles would be necessary conditions for the sustained generation of organizational-level level outcomes such as improved quality, patient safety, customer satisfaction and, distantly, population-level health outcomes. Private sector organizations outcomes may include profits, market share, efficiency and productivity gains, and customer satisfaction, among others, but these have been excluded from this study.

The framework contains three additional elements that would generate interdependence and non-linearities in the behavior of a primary care performance measurement and management system and that would help explain how performance measurement and management systems could work or not, and why. These include 1) the recursive linkages among system elements, described in Figure 1 as bidirectional arrows which will likely generate feedback effects; 2) the dynamic interaction between context and system actors, which will likely introduce context-specific variations in the outcomes from performance management interventions; and, 3) the repetition of performance measurement and management cycles as a precondition for the generation of sustained change/improvement through time.

Also, given the well-known limits to the adoption and use of evidence by healthcare system actors at all levels ^48– 53^, the use of performance information is a critical, intermediate factor in the process of production of downstream outcomes. In ways that are similar to how the results of evaluation studies may or may not be used ^54– 59^, system actors’ use of performance information is oftentimes implicit in assumptions about how performance measurement and management systems are supposed to generate multi-level outcomes. Performance information use can be defined as *“the assessments, decisions, or attitudes that primary care system actors and stakeholders hold towards the interventions that are the object of the PM system”* ^27^.

Primary care system actors’ assessments, decisions or attitudes can be triggered or not in response to 1) the performance measurement and management interventions in use; and, 2) the contextual conditions in which they are embedded ^41, 60– 64^. For performance measurement and management systems to achieve desirable effects, the supply of performance information needs to be accompanied by individual and organizational decisions to act upon it. Unfortunately, production of the former does not always guarantee the achievement of the latter ^40, 65^. Also, the assumption that adopting performance information will only have positive effects has also been proven not to be correct at all times ^66– 68^.

Context factors, or the environment or setting in which the proposed process of change is to be implemented can exert influence through interactions that occur at multiple levels (individual, interpersonal, organizational, community and societal) within the primary care system. Such factors can facilitate or inhibit the effects of performance measurement and management systems and are exemplified by the composition and dynamics of the institutional primary care setting (policies, legislation, and sector-specific reforms, among others); the degree of autonomy or flexibility granted to primary care delivery actors to innovate and implement organizational changes; and by social and political pressures for transparency, accountability or social control, among others. System antecedents, such as experiences with previous institutional reforms, and the readiness for change in the primary care system, have also been shown to have effects on the acceptance and assimilation of performance improvements ^69, 70^. Finally, ancillary components like technical assistance, monitoring and evaluation, and training, among others, should also be considered as relevant factors that can contribute or create obstacles in the generation of performance improvements ^25, 37, 44^.

## Specific Intervention and outcomes of interest

The conceptual frameworks outlined above informs the scope of this EGM. To define and describe the specific interventions and outcomes considered for inclusion, this study uses an adapted version of the taxonomy developed by the Effective Practice and Organization of Care (EPOC) ^71^. Within the general categories described in this taxonomy, the study will focus on two: 1) Implementation strategies; 2) Accountability arrangements; and, 3) Some types of financial arrangements.

Implementation strategies are defined as interventions designed to bring about changes in healthcare organization, the behavior of healthcare professionals or the use of health services by recipients ^37^. Financial arrangements refer to changes in how funds are collected, insurance schemes, how services are purchased, and the use of targeted financial incentives or disincentives ^71^. These two categories of interventions can operate at individual- (providers, managers, etc.) or organizational-levels (facilities, networks of care, local health systems, etc.).

Accountability interventions at individual, organizational, and community-levels were also included as a separate category. Given the growing interest on values like people-centered care and the confluence of the latter with long-standing interest in community participation and citizen engagement, there has been an increase in the availability of evidence surrounding the policy relevance of social accountability interventions as a system of external control that can drive performance improvements in primary care delivery systems ^72– 77^. There has also been a long-standing focus of government-driven performance reforms focused on inducing accountability among healthcare providers using internal accountability interventions such as audit and feedback, supervision, and others. For the purposes of this study, accountability arrangements are defined as the organizational and institutional arrangements used by system actors within governments to steward the delivery of public services towards increased performance.

Within the three intervention categories of implementation strategies, accountability arrangements and financial arrangements we identified fourteen different types of interventions. Table 1 summarizes these interventions, and also indicate the level at which the interventions take place. We describe each intervention category in more detail below.

**Table 1.** Interventions of relevance to the evidence gap map.

Intervention categories	Individual-level provider interventions	Organizational-level interventions	Societal, community- level interventions
Implementation strategies	Clinical practice guidelines; Reminders; In-service training; Continuous education; Supervision	Clinical incident reporting; Clinical practice guidelines in PHC facilities; Local opinion leaders; Continuous quality improvement (including lean management).	Not applicable
Accountability arrangements	Audit and feedback		Public release of performance information; Social accountabioity
Financial arrangements	Performance-based financing (Includes supply-side Results- Based Financing, Pay for Performance, and other provider incentives and rewards)	Performance-based financing (Includes supply-side Results-Based Financing, Pay for Performance, and other facility- based incentives and rewards)	Not applicable

### Implementation strategies

In this category, we identified eight interventions of relevance including: 1) **In-service training**, a form of positive behavior support aimed at increasing the capabilities of individual primary care system actors ^78^; 2) **Reminders**, manual or computerized interventions that prompt individual providers to perform an action during a clinical exchange and can include, among others, job aids, paper reminders, checklists, and computer decision support systems ^71, 79– 83^; 3) **Clinical practice guidelines**, or systematically developed statements to assist healthcare providers and patients to decide on appropriate health care for specific circumstances ^71, 84– 88^; 4) **Continuous education,** referring to courses, workshops, or other educational meetings aimed at increasing the technical competencies of primary care providers; 5) **Clinical incident reporting,** or systems for reporting critical incidents and adverse or undesirable effects as a means to improving the safety of healthcare delivery ^33^; 6) **Local opinion leaders**, referring to the identification and use of identifiable local opinion leaders to promote good clinical practices ^31, 89^; 7) **Continuous quality improvement** defined as the iterative process to review and improve care that includes involvement of healthcare teams, analysis of a process or system, a structured process improvement method or problem-solving approach, and use of data analysis to assess changes ^71^. It will include lean management as one of the approaches used to improve efficiency and quality in service provider organizations ^90– 94^; and, 8) **Supervision**, defined as routine control visits by senior primary care staff to providers and facilities ^95– 101^.

### Accountability arrangements

In this category we included the following three interventions: 1) **Audit and feedback**, defined as a summary of primary care provider or facility performance over a specified period of time, given in a written, electronic, or verbal format; such interventions can occur at individual provider as well as at organizational, facility level ^102– 107^; 2) **Public release of performance data**, defined as arrangements to inform the public about the performance of primary care providers or facilities in written or electronic formats; and, 3) **Social accountability interventions**, defined as an accountability arrangement in which community members and/or civil society organizations are involved in the monitoring of performance of primary care providers or facilities ^77^.

### Financial arrangements

There are many variations in this type intervention and contested definition among them. The interventions of interest to this study are under the general heading of Performance-Based Financing (PBF) but can also include Results-Based Financing (RBF), Pay-for-Performance (PFP), and the use of provider rewards and incentives. For precision purposes, we include the definitions developed by Musgrove ^108^ for these terms:

• **Results-based financing** refers to *any program that rewards the delivery of one or more outputs or outcomes by one or more incentives, financial or otherwise, upon verification that the agreed-upon result has actually been delivered. Incentives may be directed to service providers (supply side), program beneficiaries (demand side) or both. Payments or other rewards are not used for recurrent inputs, although there may be supplemental investment financing of some inputs, including training and equipment to enhance capacity or quality; and they are not made unless and until results or performance are satisfactory*; and,
• **Performance-based financing** is a *form of RBF distinguished by three conditions. Incentives are directed only to providers, not beneficiaries; awards are purely financial--payment is by fee for service for specified services; and payment depends explicitly on the degree to which services are of approved quality, as specified by protocols for processes or outcomes*;
• **Pay-for performance, performance-based payment and performance-based incentives** *can all be considered synonyms for RBF. Performance in these labels means the same thing as results, and payment means the same thing as financing*.

### Outcomes included in the evidence gap map

Outcomes were categorized following the guidelines developed for EPOC systematic reviews and adapted for research on performance management in primary care systems in LMIC. Relevant outcomes are therefore those that can be actionable for the intended users: research groups, funding agencies, and performance measurement and management practitioners in primary care systems in LMIC. Based on these considerations outcomes of interest will be wide in scope; can occur across short- and long-term timeframes; can be observable at various levels within a system (individual, organizational, social); and, can include desirable as well as undesirable, adverse effects. The priority-setting exercise that will follow the completion of this EGM may result in the identification of primary and secondary outcomes; at this stage, however, the study aims to scope the largest number of relevant outcomes within available operational constraints.

The main outcome categories included in this EGM are listed in Table 2. They include: 1) provider and managerial outputs and outcomes, defined as individual, provider and managerial staff effects, and exemplified by changes in workload, work morale, stress, burnout, sick leave, and staff turnover; 2) patient outcomes, defined as changes in health status or on patient health behaviours; 3) organizational outcomes, defined as organizational-level effects within and across facilities and networks of primary care such as quality of care process improvements, patient satisfaction, perceived quality of care, workforce retention, organizational culture, and unintended outcomes (gaming, shirking, shaming, data falsification, etc.); 4) population-level outputs and outcomes, defined as aggregate, health and equity effects accruing defined populations, including utilization of specific primary care services (for instance, number of antenatal care visits, institutional deliveries, etc.), coverage of services (such as the proportion of pregnant women receiving antenatal care, proportion of pregnant women delivering in facilities; coverage rate of specific vaccines), access to primary care services (for instance, waiting times), adverse health effects or harm, health equity effects, and unintended health effects; and, 5) social outcomes defined as non-health, social, economic, or cultural effects affecting defined populations, such as changes in community participation, non-health equity effects, non-health adverse effects or harm, and other unintended social outcomes. Table 2 lists each outcome category and provides examples of specific types of results within each category.

**Table 2.** Outcomes of relevance to users of the evidence gap map.

Provider and managerial outputs and outcomes	Patient outcomes	Organizational outcomes	Population health outputs and outcomes	Social outcomes
Workload Work morale Stress Burnout Sick leave Staff turnover	Health status outcomes: a) Physical health and treatment outcomes such as mortality, and morbidity; b) Psychological health and wellbeing; c) Psychosocial outcomes such as quality of life, social activities Health behaviors: adherence by patients to treatment or care plans and/or health-seeking behaviors; Unintended patient outcomes	Quality of care process improvements; Adherence to recommended practice or guidelines; Patient satisfaction Perceived quality of care Workforce retention Changes in organizational culture Unintended organizational outcomes	Utilization of specific services (example: number of antenatal visits) Coverage of specific services or interventions (example: proportion of pregnant women receiving antenatal care; proportion of pregnant women delivering in facilities; coverage rate of specific vaccines) Access to primary care services (example waiting times) Health equity effects Adverse health effects or harm Unintended population outcomes	Community participation Other equity effects Unintended social outcomes

Adapted from: Cochrane Effective Practice and Organization of Care (EPOC). What outcomes should be reported in EPOC reviews? EPOC resources for review authors, 2017.

## Methods

### Overall approach

The team will follow the methodology to produce evidence gap maps developed by 3ie ^109, 110^. The methodology was developed as a tool to systematically map evidence and research gaps on intervention effects for a broad topic area. In doing so, EGMs can help inform strategic use of resources for new research by identifying ‘absolute gaps’ where there are few or no available impact evaluations, and ‘synthesis gaps’ where there are clusters of impact evaluations but no available high-quality systematic reviews. By making existing studies easily available to researchers and describing the broad characteristics of the evidence base, the EGM can also inform the methods and design of future studies. EGMs may also facilitate the use of evidence to inform decisions by providing collections of systematic reviews that are critically appraised and ready for use by various decision makers. The methods used to develop EGMs are informed by systematic approaches to evidence synthesis and review and include key characteristics such as explicit inclusion/exclusion criteria and a systematic and transparent approach to study identification, data extraction and analysis. We describe our methods in more detail below.

### Criteria for including and excluding studies

The process starts with developing the scope for the EGM by creating an evidence-informed framework that serves for the identification of the interventions and outcomes that are relevant for the domains under study. To do so we drew on several existing frameworks (cited above) and adapted these according to the scope of our work, which had been broadly defined to focus on performance management and measurement in a primary care setting.

Table 1 and Table 2 above define the final intervention and outcome inclusion criteria. To be included, studies have to assess the effect of at least one of these interventions on one of the outcomes.

Performance management and measurement interventions are of relevance across the health sector. To make our study manageable within operational constraints, we will focus on the supply-side, of primary care service delivery. We will exclude demand-side health interventions, such as conditional cash transfers, communication for behavior-change, and social marketing, among others. We will also exclude public health interventions such as epidemiological surveillance. Finally, services delivered in hospitals will also be excluded.

### Types of included study designs

We will include studies designed to assess the effects of interventions, and systematic reviews of such studies, as defined below:

• Explicitly described as systematic reviews and reviews that describe methods used for search, data collection and synthesis as per the protocol for the 3ie database of systematic reviews (Snilstveit *et al*., 2018).
• Impact evaluations, defined as program evaluations or field experiments that use experimental or observational data to measure the effect of a program relative to a counterfactual representing what would have happened to the same group in the absence of the program. Specifically we will include the following impact evaluation designs: Randomized controlled trials (RCT) where the intervention is randomly allocated at the individual or cluster level; Regression discontinuity design (RDD); Controlled before and after studies using appropriate methods to control for selection bias and confounding such as Propensity Score Matching (PSM) or other matching methods; Instrumental Variables Estimation or other methods using an instrumental variable such as the Heckman Two Step approach; Difference-in-Differences (DD) or a fixed- or random-effects model with an interaction term between time and intervention for baseline and follow-up observations; Cross-sectional or panel studies with an intervention and comparison group using methods to control for selection bias and confounding as described above; and, Interrupted-time series (ITS), a type of study that uses observations at multiple time points before and after an intervention (the ‘interruption’). We will only include ITS studies that use at least three observations before and three observations after the intervention.

Efficacy trials and systematic reviews of efficacy trials will be excluded. Broadly, efficacy trials determine whether an intervention produces the expected result under ideal/controlled circumstances, whereas effectiveness trials measure the degree of beneficial effect under “real world” settings. However, the distinction between these two types of studies is generally considered as a continuum rather than a clear dichotomy and in practice it can be difficult to clearly categorize a trial as either effectiveness or efficacy ^111^. We will therefore draw on the criteria developed by Snilstveit ^112^ *et al*. to aid the identification of efficacy trials for exclusion from the EGM. The adapted criteria are as follows:

• Research Objective: Is the study primarily designed to determine to what extent a specific technique, technology, treatment, procedure or service works under ideal condition rather than attempt to answer a question relevant to the roll-out of a large program?
• Providers: Is the intervention primarily delivered by the research study team rather than primary health care personal/trained laypersons who don’t have extensive expertise?
• Delivery of intervention: Is the intervention delivered with high degree of assurance of delivery of the treatment? (Is the delivery tightly monitored/supervised by the researcher following specific protocols; Is adherence to the treatment monitored closely with frequent follow- ups?)

### Other inclusion and exclusion criteria

In addition, studies have to be conducted in a low- or middle-income country as defined by the World Bank. We will exclude studies exclusively focused on high-income countries, or systematic reviews focusing on a single country. Moreover, studies have to be published in any language in the year 2000 and after. Studies published before 2000 will be excluded. We will include studies regardless of status (ongoing or completed) and type of publication, published (e.g. journal article, book chapter) and unpublished (e.g. report or working paper).

### Search strategy

We have developed a systematic search strategy in collaboration with two information specialists. We developed a detailed search string for searching bibliographic databases and relevant portals. A sample strategy was developed for Medline, (see Supplementary File 1) and covers a detailed explanation of the search terms used based on an initial set of English search terms relevant to the main concepts of our inclusion criteria, including intervention, study design and population (low- and middle-income countries). These were combined using appropriate Boolean operators. All search strategies used in the study will be published along with study results.

We will identify potential studies using three strategies as listed below:

• Advanced search of the following bibliographic databases such as Medline (Ovid), Embase (Ovid), CAB Global Health (Ovid), CINAHL (Ebsco), Cochrane Library, Scopus (Elsevier), and Econlit (Ovid);
• Search of key institutional databases, repositories of impact evaluations and systematic reviews and other sources of grey literature such as the International Initiative for Impact Evaluation Impact Evaluation and Systematic Review repositories; Cochrane Effective Practice and Organization of Care (EPOC); the Abdul Latif Jameel Poverty Action Lab (J-PAL); The World Bank’s Independent Evaluation Group; Inter-American Development Bank repository; and, American Economic Association Register;
• Snowballing the references in appraised systematic reviews and citation tracking of included studies using Scopus and contacting authors, when required.

### Procedures for screening and data extraction

Following the search, we will import all records into EPPI reviewer 4. Following the removal of duplicates, we will combine manual screening and text mining to assess studies for inclusion at the title and abstract stage. To ensure consistent application of screening criteria for all screeners, we plan to assess the same random sample of 100 abstracts. Any discrepancies will be discussed within the team and inclusion criteria will be clarified if necessary. Following this initial set of 100, we will move to single screening with “safety first approach”, whereby there is an option to mark unclear studies for review by a second reviewer ^113^.

Once all screeners have been trained, we will screen a random sample of 500 abstracts to train EPPI reviewer’s priority screening function. The priority screening function can be used at the title/abstract screening stage to prioritize the items most likely to be categorized as ‘to-be included’ based on previously included documents. Using priority screening in this way allows for the identification of includable records at an earlier stage in the review process so that work can begin earlier on full-text screening and data extraction.

Depending on the number of search hits, we may also make use of EPPI reviewer’s auto-exclude function to auto-exclude studies from the search that have less than a ten per cent probability score of inclusion. This function classifies un-screened studies into ten percent intervals of probability of inclusion, based on keywords included in previously included and excluded studies.

Because of time and resource constraints we will not conduct independent double screening of all studies that will be considered at full text. To minimize bias and human error we will however double screen a sample of studies at the beginning to ensure inter-rater reliability between screeners, as described for the title/abstract stage above. In addition, we will take a “safety first” approach at the title and abstract stage, whereby any studies where the first screener is uncertain about inclusion/exclusion will be screened by a second person ^113^. All studies identified for inclusion will be effectively screened by a second/third person during data extraction.

We will use a standardized data extraction form in Microsoft Excel to systematically extract meta-data from all included studies, including bibliographic details, intervention type and description, outcome type and definition, study design, and geographical location. We will also assess the extent to which studies incorporate a consideration of equity, and extract information about if and how studies consider vulnerable and marginalized groups. To do so we will draw on the PROGRESS-Plus framework ^114^ which outlines dimensions that may give rise to inequity in either access to services, or final health outcomes. In particular, we will consider the following dimensions: Place of residence (location of household such as distance from health facility, or rural/urban), ethnicity, culture and language, gender, socioeconomic status and other vulnerable groups (open category to be used iteratively to record details of any vulnerable groups identified during coding).

For each study we will assess if they consider equity for any of these dimensions, and if so how, giving the following options: 1) Contains equity-sensitive analytical frameworks/theory of change; 2) Uses equity-sensitive research questions; 3) Follows equity-sensitive methodologies (sub-group analysis); 4) Contains equity-sensitive methodologies: additional study components to assess how and why (including mixed and qualitative methods); 5) Uses any other methodology that is equity sensitive that is not covered by the other options; 6) Uses equity-informed research processes (who are the respondents, who collects data, when, where etc.); 7) Addresses interventions targeting specific vulnerable groups - Looks at the impact of an intervention that targets specific population groups; and/or, 8) Measures effects on an inequality outcome.

For multi-arm trials testing different interventions, each comparison arm will be treated as an individual study for the coding of interventions. We will report both number of studies and number of papers identified. In addition, we will report on the number of linked studies. Studies will be considered linked if there are multiple papers by the same study team on the same impact evaluation reporting different outcomes or different follow-up periods. If they report the same information, the study will be excluded as a duplicate.

A full list of descriptive data to be extracted is included in the coding tools in Supplementary File 2. We will begin the coding process with a training with the whole research team. This training will involve coding one included systematic review and one included impact evaluation as a group to familiarize all coders with the coding tools. The entire research team will then independently pilot the coding tool on the same small subset of studies to ensure consistency in coding and to resolve any issues or ambiguities. We will start this process with two systematic reviews and two impact evaluations, and test an additional small subset if issues or discrepancies remain in the application of the tool. Data extraction will then be completed by a single coder. To minimize bias and human error we will however review the data extraction of a sample of studies.

We will follow the adapted SURE checklist, available in the 3ie systematic review database protocol for appraisal of systematic reviews. This checklist is based on the SURE Collaboration checklist for deciding how much confidence to place in the findings of a systematic review, giving systematic reviews a rating of high, medium or low confidence ( Supplementary File 3). All systematic reviews will be appraised by at least two people; a user-friendly summary will be generated for all reviews of high confidence, and the results will be shared with study authors before publication of the EGM results.

### Statistical analysis plan and EGM visualization

Upon completion of the data collection, findings will be initially presented in a visual interactive format using 3ie’s custom-built platform and accompanied by a detailed report.

The visual, online EGM will be built by, first, transforming the intervention-outcome framework into a matrix followed by the uploading of cvs files with data for all the studies included in the map (intervention, outcome, study type, impact evaluation study design, systematic review confidence level, geographical location and equity focus). This data will automatically populate the framework matrix to indicate the relative availability of evidence. This will be used to identify and describe absolute evidence gaps (no studies) and synthesis gaps (sizeable impact evaluation literature, but no high confidence SR). In addition, the map will contain descriptions of the characteristics of the evidence using graphs, figures and descriptive statistics.

## Discussion

Evidence gap maps provide collections of evidence in specific development sectors or thematic areas ^110^. They adopt systematic methods to identify and map evidence from systematic reviews and impact evaluations. They are structured around a framework of interventions and outcomes of relevance to any given sector and this is used to provide a graphical display of the volume of ongoing and completed impact evaluations and systematic reviews, with a rating of the confidence in the findings from systematic reviews. EGMs can be used to identify areas where there is a need for the generation of new or more rigorous research evidence; also, to inform decisions by policymakers and development practitioners as policies and programs are designed.

Current best -practice in the design of evidence gap maps recommends that EGMs have a pre-specified protocol, have a systematic search strategy, contain precise and clear criteria for inclusion and exclusion, and systematically report all eligible studies. This protocol is the first step in ensuring compliance with such practices. As the visual interface is built, a final report will be submitted for peer-reviewed publication and will include a summary of the findings from the evidence gap analysis and recommendations for a prioritized research agenda on performance measurement and management in LMIC.

This evidence gap map aims to identify and describe the existing evidence on the effects of interventions in the area of performance measurement and management in primary care delivery systems in LMICs and, also, to provide easy access to the best available existing evidence on intervention effects in this area. As a result, the EGM will inform the development of a prioritized research agenda for primary care delivery systems in LMICs.

## Dissemination of findings

Findings will be presented at the 5 ^th^ Global Health System Symposium in October, 2018 after which a paper will be submitted for peer-reviewed publication. We will also publish the results in the form of an interactive Evidence Gap Map, which will be made freely available from the 3ie website. We will use our institutional channels to disseminate our findings as widely as possible, including via our websites, social media platforms and events beyond the Global Health System Symposium.

## Study status

By the time of submission of this paper, the framework, search strategies and data extraction tools included in this protocol have been completed. Data collection, analysis and development of the graphical interface will be completed by September 2018. A paper summarizing study results and implications will be submitted by December, 2018.

## Data availability

No data is associated with this article.

## Supplementary material

**Supplementary File 1** – Medline search strategy

.

**Supplementary File 2** – Coding tools containing full list of descriptive data to be extracted

.

**Supplementary File 3** – Systematic Reviews critical appraisal checklist
