# Supplementary material for: Evidence gap map of performance measurement and management in primary care delivery systems in low- and middle-income countries – Study protocol
Source: Gates Open Res. 2018 Nov 2;2:27. Originally published 2018 May 29. [Version 2] doi: 10.12688/gatesopenres.12826.2 (PMC6030397; doi:10.12688/gatesopenres.12826.2)
Supplement: Supplementary file 1 [file gatesopenres-2-13958-s0000.tgz › a89581eb-1ea5-49f7-882b-f91aabfa3cd3.docx]

**Supplementary file 1 – Medline Search Strategy**

| \|  \| \| **Search** \| **Results** \| \| --- \| --- \| --- \| --- \| \| 1 \| \| Community Mental Health Services/ or Family Practice/ or Home Care Services/ or Physicians, Family/ or Community Health Services/ or Community Health Nursing/ or Community Pharmacy Services/ or Community Health Workers/ or Preventive Health Services/ or Primary Health Care/ or Rural Health/ or Health Promotion/ \| 317310 \| \| 2 \| \| (Primary care or General practi* or Primary health* or Community mental health* or Family practice or Family medicine or Family physician* or Home care or Home based or Home health* or Community health* or Community nurs* or health visit* or Community pharmac* or Preventive care or Prevention program* or Preventive service* or Preventive health).ab,kw,ti. \| 284477 \| \| 3 \| \| exp Nurses/ or Nurse Anesthetists/ or Nurse Clinicians/ or Nurse Midwives/ or Family Nurse Practitioners/ or Nurses, International/ or Nurses, Male/ or Nursing Staff/ or Nurses' Aides/ or Students, Nursing/ or midwifery/ or Nurse Practitioners/ \| 138739 \| \| 4 \| \| (nurse* or nursing auxiliar* or nursing assistant* or midwife* or midwives).ab,kw,ti. \| 258722 \| \| 5 \| \| (paraprofessional* or paramedic* or paramedical or allied health personnel or allied health worker* or support worker* or home health aide* or trained volunteer* or ((trained or lay or community or village or maternal or rural) adj3 (health volunteer* or health worker* or healthcare worker* or health care worker* or nutrition worker* or health agent* or health guide* or health visitor* or health advocate* or health promoter*)) or treatment supporter* or birth attendant* or TBA* or shasthyo sebika or agente communitario de saude or visitador* or women group leader* or accompagnateur* or saksham sahaya or anganwadi worker* or behvarz or brigadistas or lady health worker* or trained mother* or community drug distributor* or (lay adj (volunteer* or worker* or visitor* or attendant* or aide* or support* or person* or helper* or caregiver* or consultant* or assistant* or staff))).ab,kw,ti. \| 32769 \| \| 6 \| \| ((lay or voluntary or volunteer? or untrained or unlicensed or nonprofessional? or non professional?) adj5 (worker? or visitor? or attendant? or aide or aides or support$ or person* or helper? or carer? or caregiver? or care giver? or consultant? or assistant? or staff or visit$ or midwif* or midwiv*)).ab,kw,ti. \| 8106 \| \| 7 \| \| (community adj3 (health worker? or health care worker? or healthcare worker? or health or worker* or nurs* or aide* or volunteer? or support)).ab,kw,ti. \| 50067 \| \| 8 \| \| 1 or 2 or 3 or 4 or 5 or 6 or 7 \| 798753 \| \| 9 \| \| ((match* adj3 (propensity or coarsened or covariate)) or "propensity score" or ("difference in difference*" or "difference-in-difference*" or "differences in difference*" or "differences-in-difference*" or "double difference*") or ("quasi-experimental" or "quasi experimental" or "quasi-experiment" or "quasi experiment") or ((estimator or counterfactual) and evaluation*) or ("instrumental variable*" or (IV adj2 (estimation or approach))) or "regression discontinuity").ab,kw,ti. \| 28174 \| \| 10 \| \| (((experiment or experimental) adj2 (design or study or research or evaluation or evidence)) or (random* adj4 (trial or assignment or treatment or control or intervention* or allocat*))).ab,kw,ti. \| 379189 \| \| 11 \| \| Randomized Controlled Trial/ or Randomized Controlled Trials as Topic/ or random allocation/ or Propensity Score/ or Models, Econometric/ or Quasi-Experimental Studies/ or controlled before-after studies/ or cross-sectional studies/ or Regression Analysis/ \| 1026188 \| \| 12 \| \| Program Evaluation/ or Evaluation Studies/ \| 283871 \| \| 13 \| \| ((impact adj2 (evaluat* or assess* or analy* or estimat* or measure)) or (effectiveness adj2 (evaluat* or assess* or analy* or estimat* or measure))).ab,kw,ti. \| 133624 \| \| 14 \| \| ("program* evaluation" or "project evaluation" or "evaluation research" or "natural experiment*" or "program* effectiveness").ab,kw,ti. \| 10392 \| \| 15 \| \| Interrupted Time Series Analysis/ \| 434 \| \| 16 \| \| "Interrupted Time Series".ab,kw,ti. \| 2030 \| \| 17 \| \| Meta-Analysis/ \| 88665 \| \| 18 \| \| ((systematic* adj2 review*) or "systematic review" or "meta-analy*" or "meta analy*" or metaanaly*).ab,kw,ti. \| 202721 \| \| 19 \| \| 9 or 10 or 11 or 12 or 13 or 14 or 15 or 16 or 17 or 18 \| 1759691 \| \| 20 \| \| Developing Countries/ \| 70087 \| \| 21 \| \| developing countries.kf,sh. \| 80984 \| \| 22 \| \| Africa/ or Asia/ or Caribbean/ or West Indies/ or South America/ or Latin America/ or Central America/ \| 70760 \| \| 23 \| \| (Africa or Asia or Caribbean or West Indies or South America or Latin America or Central America).tw. \| 157958 \| \| 24 \| \| (Afghanistan or Albania or Algeria or Angola or Argentina or Armenia or Armenian or Azerbaijan or Bangladesh or Benin or Byelarus or Byelorussian or Belarus or Belorussian or Belorussia or Belize or Bhutan or Bolivia or Bosnia or Herzegovina or Hercegovina or Botswana or Brazil or Bulgaria or Burkina Faso or Burkina Fasso or Upper Volta or Burundi or Urundi or Cambodia or Khmer Republic or Kampuchea or Cameroon or Cameroons or Cameron or Camerons or Cape Verde or Central African Republic or Chad or China or Colombia or Comoros or Comoro Islands or Comores or Mayotte or Congo or Zaire or Costa Rica or Cote d'Ivoire or Ivory Coast or Cuba or Djibouti or French Somaliland or Dominica or Dominican Republic or East Timor or East Timur or Timor Leste or Ecuador or Egypt or United Arab Republic or El Salvador or Eritrea or Ethiopia or Fiji or Gabon or Gabonese Republic or Gambia or Gaza or Georgia Republic or Georgian Republic or Ghana or Grenada or Guatemala or Guinea or Guiana or Guyana or Haiti or Honduras or India or Maldives or Indonesia or Iran or Iraq or Jamaica or Jordan or Kazakhstan or Kazakh or Kenya or Kiribati or Korea or Kosovo or Kyrgyzstan or Kirghizia or Kyrgyz Republic or Kirghiz or Kirgizstan or Lao PDR or Laos or Lebanon or Lesotho or Basutoland or Liberia or Libya or Macedonia or Madagascar or Malagasy Republic or Malaysia or Malaya or Malay or Sabah or Sarawak or Malawi or Mali or Marshall Islands or Mauritania or Mauritius or Agalega Islands or Mexico or Micronesia or Middle East or Moldova or Moldovia or Moldovian or Mongolia or Montenegro or Morocco or Ifni or Mozambique or Myanmar or Myanma or Burma or Namibia or Nepal or Netherlands Antilles or Nicaragua or Niger or Nigeria or Muscat or Pakistan or Palau or Palestine or Panama or Paraguay or Peru or Philippines or Philipines or Phillipines or Phillippines or Papua New Guinea or Romania or Rumania or Roumania or Rwanda or Ruanda or Saint Lucia or St Lucia or Saint Vincent or St Vincent or Grenadines or Samoa or Samoan Islands or Navigator Island or Navigator Islands or Sao Tome or Senegal or Serbia or Montenegro or Seychelles or Sierra Leone or Sri Lanka or Solomon Islands or Somalia or Sudan or Suriname or Surinam or Swaziland or South Africa or Syria or Tajikistan or Tadzhikistan or Tadjikistan or Tadzhik or Tanzania or Thailand or Togo or Togolese Republic or Tonga or Tunisia or Turkey or Turkmenistan or Turkmen or Uganda or Ukraine or Uzbekistan or Uzbek or Vanuatu or New Hebrides or Venezuela or Vietnam or Viet Nam or West Bank or Yemen or Zambia or Zimbabwe).ab,kw,ti. \| 894470 \| \| 25 \| \| exp africa/ or exp africa, northern/ or algeria/ or egypt/ or libya/ or morocco/ or tunisia/ or exp "africa south of the sahara"/ or africa, central/ or cameroon/ or central african republic/ or chad/ or congo/ or "democratic republic of the congo"/ or equatorial guinea/ or gabon/ or africa, eastern/ or burundi/ or djibouti/ or eritrea/ or ethiopia/ or kenya/ or rwanda/ or somalia/ or south sudan/ or sudan/ or tanzania/ or uganda/ or africa, southern/ or angola/ or botswana/ or lesotho/ or malawi/ or mozambique/ or namibia/ or south africa/ or swaziland/ or zambia/ or zimbabwe/ or africa, western/ or benin/ or burkina faso/ or cape verde/ or cote d'ivoire/ or gambia/ or ghana/ or guinea/ or guinea-bissau/ or liberia/ or mali/ or mauritania/ or niger/ or nigeria/ or senegal/ or sierra leone/ or togo/ or americas/ or exp caribbean region/ or exp west indies/ or exp central america/ or belize/ or costa rica/ or el salvador/ or guatemala/ or honduras/ or nicaragua/ or panama/ or panama canal zone/ or latin america/ or mexico/ or exp south america/ or argentina/ or bolivia/ or brazil/ or chile/ or colombia/ or ecuador/ or french guiana/ or guyana/ or paraguay/ or peru/ or suriname/ or uruguay/ or venezuela/ or asia/ or asia, central/ or kazakhstan/ or kyrgyzstan/ or tajikistan/ or turkmenistan/ or uzbekistan/ or exp asia, southeastern/ or borneo/ or brunei/ or cambodia/ or timor-leste/ or indonesia/ or laos/ or malaysia/ or mekong valley/ or myanmar/ or philippines/ or singapore/ or thailand/ or vietnam/ or asia, western/ or bangladesh/ or bhutan/ or india/ or sikkim/ or middle east/ or afghanistan/ or bahrain/ or iran/ or iraq/ or israel/ or jordan/ or kuwait/ or lebanon/ or oman/ or qatar/ or saudi arabia/ or syria/ or turkey/ or united arab emirates/ or yemen/ or nepal/ or pakistan/ or sri lanka/ or far east/ or china/ or beijing/ or macau/ or tibet/ or korea/ or mongolia/ or taiwan/ or indian ocean islands/ or comoros/ or madagascar/ or mauritius/ or reunion/ or seychelles/ or pacific islands/ or exp melanesia/ or exp micronesia/ or polynesia/ or pitcairn island/ or exp samoa/ or tonga/ or prince edward island/ or west indies/ or "antigua and barbuda"/ or bahamas/ or barbados/ or cuba/ or dominica/ or dominican republic/ or grenada/ or guadeloupe/ or haiti/ or jamaica/ or martinique/ or netherlands antilles/ or puerto rico/ or "saint kitts and nevis"/ or saint lucia/ or "saint vincent and the grenadines"/ or "trinidad and tobago"/ or united states virgin islands/ or oceania/ \| 967404 \| \| 26 \| \| ((developing or less* developed or under developed or underdeveloped or Middle income or low* income or underserved or under served or deprived or poor*) adj (countr* or nation? or population? or world or state*)).ab,kw,ti. \| 82082 \| \| 27 \| \| ((developing or less* developed or under developed or underdeveloped or middle income or low* income) adj (economy or economies or population*)).ab,kw,ti. \| 1943 \| \| 28 \| \| (low* adj (gdp or gnp or gross domestic or gross national)).tw. \| 214 \| \| 29 \| \| (low adj3 middle adj3 countr*).tw. \| 9962 \| \| 30 \| \| (lmic or lmics or third world or lami countr*).tw. \| 5339 \| \| 31 \| \| "transitional countr*".tw. \| 143 \| \| 32 \| \| 20 or 21 or 22 or 23 or 24 or 25 or 26 or 27 or 28 or 29 or 30 or 31 \| 1453535 \| \| 33 \| \| exp Inservice Training/ or Mentoring/ or Staff Development/ \| 27896 \| \| 34 \| \| ((inservice or in-service) adj2 (train* or course? or development or education or teach*)).ab,ti. \| 2532 \| \| 35 \| \| On the job training.ab,ti. \| 489 \| \| 36 \| \| 33 or 34 or 35 \| 29857 \| \| 37 \| \| (guideline* adj3 (implement* or uptake* or adopt* or adhere* or concord* or complian* or comply or non-adhere* or nonadhere* or non-concord* or nonconcord* or non-complian* or noncomplian*)).ab,ti. \| 16565 \| \| 38 \| \| (standard? adj3 (implement* or uptake* or adopt* or adhere* or concord* or complian* or comply or non-adhere* or nonadhere* or non-concord* or nonconcord* or non-complian* or noncomplian*)).ab,ti. \| 8760 \| \| 39 \| \| (national adj3 (guideline*1 or guidance or standard*1 or pathway*1 or protocol*1)).ab,ti. \| 18566 \| \| 40 \| \| ((ministry or ministries or government*?) adj3 (guideline* or guidance or standard*1 or pathway*1 or protocol*1)).ab,ti. \| 1073 \| \| 41 \| \| practice guideline/ \| 23420 \| \| 42 \| \| Practice Guidelines as Topic/ \| 103918 \| \| 43 \| \| (practice adj3 (guideline*1 or guidance or standard*1 or pathway*1)).ab,ti. \| 34503 \| \| 44 \| \| (clinical adj3 (guideline*1 or guidance or standard*1 or pathway*1 or protocol*1)).ab,ti. \| 57300 \| \| 45 \| \| (guideline* adj3 (impact or influenc* or effect* or disseminat*)).ab,ti. \| 5006 \| \| 46 \| \| (impact and guideline?).ab,ti. \| 21455 \| \| 47 \| \| (effect$ and guideline?).ab,ti. \| 74369 \| \| 48 \| \| (guideline? and disseminat$).ab,ti. \| 4531 \| \| 49 \| \| Hand Disinfection/st [Standards] \| 963 \| \| 50 \| \| Professional-Patient Relations/ \| 25018 \| \| 51 \| \| Professional-Patient Relations/es [Ethics] \| 612 \| \| 52 \| \| 37 or 38 or 39 or 40 or 41 or 42 or 43 or 44 or 45 or 46 or 47 or 48 or 49 or 50 or 51 \| 299626 \| \| 53 \| \| Reminder Systems/ \| 3073 \| \| 54 \| \| Reminder Systems/cl, st [Classification, Standards] \| 113 \| \| 55 \| \| "OUTCOME ASSESSMENT (HEALTH CARE)"/ \| 63668 \| \| 56 \| \| reminder$.tw. \| 10171 \| \| 57 \| \| (reminder$ or promt$ or cue).tw. \| 33970 \| \| 58 \| \| ((paper adj2 remind$) or (manual* adj2 remind$)).tw. \| 63 \| \| 59 \| \| 53 or 54 or 55 or 56 or 57 or 58 \| 98923 \| \| 60 \| \| (professional adj2 (development$ or education$ or retrain$ or skill? enhanc$ or (skill? adj2 improv$) or training or upgrade? or upgrading)).ab,ti. \| 14972 \| \| 61 \| \| Education, Continuing/ or Professional Education/ or Education, Public Health Professional/ or Continuing Nursing Education/ or Continuing Medical Education/ \| 57198 \| \| 62 \| \| ((education$ or train$) adj (program$ or intervention? or meeting? or session? or strateg$ or workshop?)).ab,ti. \| 97078 \| \| 63 \| \| ((education$ or train$) adj (lecture? or symposi$ or course?)).ab,ti. \| 6710 \| \| 64 \| \| 60 or 61 or 62 or 63 \| 164007 \| \| 65 \| \| exp Risk Management/ \| 262403 \| \| 66 \| \| exp Medical Errors/ \| 103335 \| \| 67 \| \| exp SAFETY/ \| 71051 \| \| 68 \| \| DISCLOSURE/ \| 12426 \| \| 69 \| \| (report* adj3 (incident* or adverse event* or voluntary or mandatory or error* or whistleblow* or whistle blow* or compulsory or error)).ab,ti. \| 25624 \| \| 70 \| \| Incident Report$.tw. \| 1801 \| \| 71 \| \| adverse event report$.tw. \| 1884 \| \| 72 \| \| voluntary report$.tw. \| 435 \| \| 73 \| \| mandatory report$.tw. \| 792 \| \| 74 \| \| error report$.tw. \| 688 \| \| 75 \| \| 65 or 66 or 67 or 68 or 69 or 70 or 71 or 72 or 73 or 74 \| 433851 \| \| 76 \| \| ((total quality or quality assurance or quality improvement) adj3 lean).ab,ti. \| 50 \| \| 77 \| \| (think lean or lean thinking).ab,ti. \| 113 \| \| 78 \| \| (lean adj3 (workflow? or efficienc$ or efficient$)).ab,ti. \| 116 \| \| 79 \| \| (lean adj3 (approach or business model? or care or collaborat$ or design$ or enterpri?e or healthcare or health care or implementation? or industry or initiative? or intervention$ or leader$ or management or methodolog$ or method? or organi?ation$ or plan or planning or philosophy or practice or practices or principles or principle or process improvement? or production or program? or programme or programmes or quality or redesign$ or reengineer$ or restructur$ or reorgani$ or safety or sigma or strategy or strategies or thinking or tool or tools or workshop$)).ab,ti. \| 1564 \| \| 80 \| \| ((PDSA or PDCA or TQIS) adj3 (cycle or process or processes or intervention or quality or lean or improv$)).ab,ti. \| 198 \| \| 81 \| \| ((PDSA or PDCA or TQIS) adj3 ("plan do study" or "plan do check")).ab,ti. \| 265 \| \| 82 \| \| 76 or 77 or 78 or 79 or 80 or 81 \| 1983 \| \| 83 \| \| Clinical audit/ or medical audit/ or nursing audit/ \| 20557 \| \| 84 \| \| (review* or meeting? or enquir* or inquir*).ab,ti. \| 1858864 \| \| 85 \| \| (confidential enquir* or confidential inquir*).ab,ti. \| 596 \| \| 86 \| \| (Verbal autops* or social autops*).ab,ti. \| 867 \| \| 87 \| \| Management Audit/ \| 2480 \| \| 88 \| \| Benchmarking/ \| 12127 \| \| 89 \| \| "benchmark*".ab,ti. \| 30264 \| \| 90 \| \| FEEDBACK, PHYSIOLOGICAL/ or FEEDBACK/ \| 36722 \| \| 91 \| \| "utilization review"/ or "concurrent review"/ or "drug utilization review"/ \| 11683 \| \| 92 \| \| (audit or audits or auditing or feedback).tw. \| 144840 \| \| 93 \| \| 83 or 84 or 85 or 86 or 87 or 88 or 89 or 90 or 91 or 92 \| 2047378 \| \| 94 \| \| Information Dissemination/ \| 14339 \| \| 95 \| \| Consumer Health Information/mt [Methods] \| 640 \| \| 96 \| \| Consumer Behavior/ \| 19748 \| \| 97 \| \| Quality Assurance, Health Care/mt [Methods] \| 6199 \| \| 98 \| \| (information adj3 (quality or public or consumer or patient)).tw. \| 27696 \| \| 99 \| \| (performance adj (indicator? or measure? or data or rating or report)).tw. \| 13288 \| \| 100 \| \| (quality adj2 (report? or public report? or information or benchmark? or indicator?)).tw. \| 14379 \| \| 101 \| \| 94 or 95 or 96 or 97 or 98 or 99 or 100 \| 89067 \| \| 102 \| \| Motivation/ or Goals/ \| 73288 \| \| 103 \| \| Staff Development/ or Social Support/ or Peer Group/ or inservice training/ or staff development/ \| 105859 \| \| 104 \| \| (education or training or lecture? or workshop? or workshop? or mentoring or coaching or counseling or counselling or feedback or feed back or supervis*).ab,ti. \| 900935 \| \| 105 \| \| (financial support or economic support or monetary support or financial drive* or economic drive* or monetary drive* or reward* or income or pension? or remuneration or salary or salaries or wage or wages or fringe benefit? or compensation or pay for performance or health insurance? or benefit plan? or payment? or loan? or bursary or bursaries or bonus or bonuses or stipend* or honorarium or gift? or token? or allowance?).ab,ti. \| 259354 \| \| 106 \| \| REIMBURSEMENT, INCENTIVE/ \| 3941 \| \| 107 \| \| "Salaries and Fringe Benefits"/ \| 14971 \| \| 108 \| \| Physician Incentive Plans/ or Employee Incentive Plans/ \| 3691 \| \| 109 \| \| REIMBURSEMENT, INCENTIVE/ \| 3941 \| \| 110 \| \| (conditional adj3 (pay$ or transfer?)).tw. \| 296 \| \| 111 \| \| (pay$ adj3 performance).tw. \| 2213 \| \| 112 \| \| ((incentive? or compensatory or reimbursement) adj plan?).tw. \| 220 \| \| 113 \| \| result? based.tw. \| 11027 \| \| 114 \| \| performance based.tw. \| 4187 \| \| 115 \| \| ((result? or performance or output or out put) adj2 (financ$ or fund$ or pay$ or disburs$ or fee? or incentive? or initiative? or contract? or aid)).tw. \| 9347 \| \| 116 \| \| (payment incentive? or monetary incentive? or economic incentive? or financial incentive? or reimbursement incentive?).tw. \| 5660 \| \| 117 \| \| 102 or 103 or 104 or 105 or 106 or 107 or 108 or 109 or 110 or 111 or 112 or 113 or 114 or 115 or 116 \| 1275398 \| \| 118 \| \| "opinion leader*".tw. \| 1185 \| \| 119 \| \| (stakeholder or "opinion leader" or "community leader*" or "community of practice").tw. \| 11449 \| \| 120 \| \| ((stakehold* or leader*) adj2 (opinion* or organiz* or administ* or physician* or nurs* or "health professionals" or communit* or servic* or patient* or health or medical)).tw. \| 15777 \| \| 121 \| \| exp LEADERSHIP/ \| 37003 \| \| 122 \| \| 118 or 119 or 120 or 121 \| 56415 \| \| 123 \| \| (patient adj supervis$).tw. \| 54 \| \| 124 \| \| Patient Care Management/og, st [Organization & Administration, Standards] \| 1196 \| \| 125 \| \| 123 or 124 \| 1250 \| \| 126 \| \| 36 or 52 or 59 or 64 or 75 or 82 or 93 or 101 or 117 or 122 or 125 \| 3750553 \| \| 127 \| \| 8 and 19 and 32 and 126 \| 13989 \| \| 128 \| \| limit 127 to (humans and yr="2000 -Current") \| 11735 \| \|  \| \|  \|  \| \|  \| |
| --- | --- | --- | --- | --- | --- | --- | --- | --- | --- | --- | --- | --- | --- | --- | --- | --- | --- | --- | --- | --- | --- | --- | --- | --- | --- | --- | --- | --- | --- | --- | --- | --- | --- | --- | --- | --- | --- | --- | --- | --- | --- | --- | --- | --- | --- | --- | --- | --- | --- | --- | --- | --- | --- | --- | --- | --- | --- | --- | --- | --- | --- | --- | --- | --- | --- | --- | --- | --- | --- | --- | --- | --- | --- | --- | --- | --- | --- | --- | --- | --- | --- | --- | --- | --- | --- | --- | --- | --- | --- | --- | --- | --- | --- | --- | --- | --- | --- | --- | --- | --- | --- | --- | --- | --- | --- | --- | --- | --- | --- | --- | --- | --- | --- | --- | --- | --- | --- | --- | --- | --- | --- | --- | --- | --- | --- | --- | --- | --- | --- | --- | --- | --- | --- | --- | --- | --- | --- | --- | --- | --- | --- | --- | --- | --- | --- | --- | --- | --- | --- | --- | --- | --- | --- | --- | --- | --- | --- | --- | --- | --- | --- | --- | --- | --- | --- | --- | --- | --- | --- | --- | --- | --- | --- | --- | --- | --- | --- | --- | --- | --- | --- | --- | --- | --- | --- | --- | --- | --- | --- | --- | --- | --- | --- | --- | --- | --- | --- | --- | --- | --- | --- | --- | --- | --- | --- | --- | --- | --- | --- | --- | --- | --- | --- | --- | --- | --- | --- | --- | --- | --- | --- | --- | --- | --- | --- | --- | --- | --- | --- | --- | --- | --- | --- | --- | --- | --- | --- | --- | --- | --- | --- | --- | --- | --- | --- | --- | --- | --- | --- | --- | --- | --- | --- | --- | --- | --- | --- | --- | --- | --- | --- | --- | --- | --- | --- | --- | --- | --- | --- | --- | --- | --- | --- | --- | --- | --- | --- | --- | --- | --- | --- | --- | --- | --- | --- | --- | --- | --- | --- | --- | --- | --- | --- | --- | --- | --- | --- | --- | --- | --- | --- | --- | --- | --- | --- | --- | --- | --- | --- | --- | --- | --- | --- | --- | --- | --- | --- | --- | --- | --- | --- | --- | --- | --- | --- | --- | --- | --- | --- | --- | --- | --- | --- | --- | --- | --- | --- | --- | --- | --- | --- | --- | --- | --- | --- | --- | --- | --- | --- | --- | --- | --- | --- | --- | --- | --- | --- | --- | --- | --- | --- | --- | --- | --- | --- | --- | --- | --- | --- | --- | --- | --- | --- | --- | --- | --- | --- | --- | --- | --- | --- | --- | --- | --- | --- | --- | --- | --- | --- | --- | --- | --- | --- | --- | --- | --- | --- | --- | --- | --- | --- | --- | --- | --- | --- | --- | --- | --- | --- | --- | --- | --- | --- | --- | --- | --- | --- | --- | --- | --- | --- | --- | --- | --- | --- | --- | --- | --- | --- | --- | --- | --- | --- | --- | --- | --- | --- | --- | --- | --- | --- | --- | --- | --- | --- | --- | --- | --- | --- | --- | --- | --- | --- | --- | --- | --- | --- | --- | --- | --- | --- | --- | --- | --- | --- | --- | --- | --- | --- | --- | --- | --- | --- | --- | --- | --- | --- | --- | --- | --- | --- | --- | --- | --- | --- | --- | --- | --- | --- | --- | --- | --- | --- | --- | --- | --- | --- | --- | --- | --- | --- | --- | --- | --- | --- | --- | --- | --- | --- | --- | --- | --- | --- | --- | --- | --- | --- | --- | --- | --- | --- |
