# Supplementary material for: Evidence gap map of performance measurement and management in primary care delivery systems in low- and middle-income countries – Study protocol
Source: Gates Open Res. 2018 Nov 2;2:27. Originally published 2018 May 29. [Version 2] doi: 10.12688/gatesopenres.12826.2 (PMC6030397; doi:10.12688/gatesopenres.12826.2)
Supplement: Supplementary file 2 [file gatesopenres-2-13958-s0001.tgz › 21fbeb04-8206-4245-9184-d0d1a7acd962_supp_file_2.docx]

**Supplementary file 2 – coding tools containing full list of descriptive data to be extracted**

**Data extraction tool for Performance Measurement and Management (PMM) impact evaluations**

| **Category** | | **Answer** |
| --- | --- | --- |
| **Descriptive information** | ID | Open answer – xxx |
|  | Title | Open answer – this is the title that will be displayed on the EGM platform |
|  | Full title | Open answer – this is the title of the paper |
|  | Author Citation | Open answer |
|  | Publication date | Open answer |
|  | Map | Performance measurement and management in primary care systems in low and middle-income countries evidence gap map |
|  | Regions (3ie) | - East Asia and Pacific - South Asia - Europe - CIS - Middle East and North Africa - Sub-Saharan Africa - Latin America and the Caribbean |
|  | Country | See relevant country list |
|  | Study design (broad) | - Randomized Controlled Trial (RCT) - Regression Discontinuity Design (RDD) - Controlled-Before and After Study (Treatment and Comparison Group, Baseline and Endline Data) - Panel data but no Baseline (Treatment and Comparison Group, Panel Data but no Baseline Data) - Comparison Group with Endline Data Only - Interrupted Time Series (ITS) - Other |
|  | Study design / analysis | - Difference-in-Differences (DID) Estimation Methods - Fixed- or random effects model - Instrumental Variables (IV) - Propensity Score Matching (PSM) and other matching - Multivariate Regression - Comparison of Means - Other |
|  | Comments on study design | Open answer – any relevant information on the impact evaluation study design and analysis methods – for example if methods are combined or the authors use multiple methods separately. |
|  | Mixed methods? | Yes/No  This refers to whether or not the paper includes a qualitative component . |
| **Intervention** | Category of Intervention | - Refer to table 1 |
|  | Interventions | - Refer to table 1 |
|  | Intervention includes multiple PMM-components | Yes/No |
|  | Intervention includes non-PMM component? | Yes/No |
|  | Intervention(s) description | Open answer - This refers to the intervention description given by the review authors rather than the descriptions of interventions in each impact evaluation included in the EGM. This should be a succinct summary of the intervention – including a description of whether the intervention is a single intervention from the framework or a package of interventions. |
| **Outcomes**  N.B. For the definitions, refer to the outcome definitions given by the review authors rather than the descriptions of outcomes in each primary study included in the review. Applies to each outcome definition question for SRs. | Outcomes | - Refer to table 2. |
|  | Provider and managerial outputs and outcomes (effects at the level of individual providers and managerial staff) | Open answer, refers to the following outcomes:   - Workload - Work morale - Stress - Burnout - Sick leave - Staff turnover - Knowledge - Attitudes, beliefs, perception - Skills and competencies |
|  | Patient outcomes (defined as changes in health status and/or on patient health behaviors) | Open answer, refers to the following outcomes:   - Health Status Outcomes: (1) Physical health - Health Status Outcomes: (2) Psychological health - Health Status: (3) Psychosocial health - Health Behaviors: (1) Adherence by patients - Health Behaviors: (2) Health seeking behaviors - Unintended patient outcomes |
|  | Organizational outcomes (defined as organizational-level within and across facilities and networks of primary care) | Open answer, refers to the following outcomes:   - Quality of care process improvement; - Adherence to recommended practice or guidelines - Patient satisfaction - Perceived quality of care - Workforce retention - Changes in organizational culture - Unintended organizational outcomes (gaming, shirking, shaming, data falsification, etc.) |
|  | Population health outputs and outcomes (defined as aggregate, health and equity effects accruing defined populations) | Open answer, refers to the following outcomes:   - Utilization of specific primary care services; - Coverage of specific services or interventions (example: proportion of pregnant women receiving antenatal care; proportion of pregnant women delivering in facilities; coverage rate of specific vaccines); - Access to primary care services (example waiting times) - Health equity effects - Adverse health effects or harm - Unintended population health outcomes |
|  | Social outcomes (defined as non-health, social, economic, or cultural effects affecting defined populations) | Open answer, refers to the following outcomes:   - Community participation - Other equity effects - Other unintended social outcomes |
| **Equity Data** | How does this study consider equity? | - Does not address equity - Contains equity-sensitive analytical frameworks / theory of change - Uses equity-sensitive research questions - Follows equity-sensitive methodologies (sub-group analysis) - Contains equity-sensitive methodologies: additional study components to assess how and why (including mixed and qualitative methods) - Uses any other methodology that is equity sensitive that is not covered by the other options - Uses equity-informed research processes (who are the respondents, who collects data, when, where etc.) - Addresses interventions targeting specific vulnerable groups - Looks at the impact of an intervention that targets specific population groups - Measures effects on an inequality outcome |
|  | Dimension of equity/ Population group | - Age - Disability - Education - Gender, sex - Occupation - Other vulnerable groups - Place of residence - Race, ethnicity, culture and language - Religion - Social capital - Socioeconomic status |
|  | Equity description | Open answer – provide a description of how the study considers equity, and for which group. This is to corroborate and elaborate on the answers above. For example, describe the sub-group analysis undertaken, how the intervention targets a disadvantaged group or how the authors used an equity sensitive framework to inform their study. Please also note the page number where this information can be found. |
| **Access** | Link | Open answer (if already on 3ie database, please use this link) |
|  | On 3ie database (yet)? | Yes/No |
|  | Publication status | - Journal Article - Published Working Paper - Book or Book Chapter - Conference Paper - Dissertation - Published Report - Unpublished Paper |

**Data extraction tool for Performance Measurement and Management (PMM) systematic reviews (SR)**

| **Category** | | **Answer** |
| --- | --- | --- |
| **Descriptive information** | ID | Open answer - SRxxx |
|  | Title | Open answer |
|  | Full title | Open answer |
|  | Author Citation | Open answer |
|  | Publication date | Open answer |
|  | Maps | Performance measurement and management in primary care systems in low and middle-income countries evidence gap map |
|  | Regions (3ie) | - East Asia and Pacific - South Asia - Europe - CIS - Middle East and North Africa - Sub-Saharan Africa - Latin America and the Caribbean - North America - Global - Unclear |
|  | SR includes studies from high-income countries? | Yes/No |
|  | Number of impact evaluations included (Systematic review only) | Open answer |
| **Intervention** | Category of Intervention | - Refer to Table 1 |
| **Intervention/ outcome**  *N.B. This refers to the intervention description given by the review authors rather than the descriptions of interventions in each primary study included in the review.* | Interventions | - Refer to Table 1 |
|  | Intervention(s) description | Open answer. |
|  | SR covers non-PMM interventions | Yes/No |
|  | Outcomes | Refer to Table 2 in the protocol |
|  | Provider and managerial outputs and outcomes (effects at the level of individual providers and managerial staff) | Open answer, refers to the following outcomes:   - Workload - Work morale - Stress - Burnout - Sick leave - Staff turnover - Knowledge - Attitudes, beliefs, perception - Skills and competencies |
| **Outcome definitions** | Patient outcomes (defined as changes in health status and/or on patient health behaviors) | Open answer, refers to the following outcomes:   - Health Status Outcomes: (1) Physical health - Health Status Outcomes: (2) Psychological health - Health Status: (3) Psychosocial health - Health Behaviors: (1) Adherence by patients - Health Behaviors: (2) Health seeking behaviors   Unintended patient outcomes |
|  | Organizational outcomes (defined as organizational-level within and across facilities and networks of primary care) | Open answer, refers to the following outcomes:   - Quality of care process improvement; - Adherence to recommended practice or guidelines - Patient satisfaction - Perceived quality of care - Workforce retention - Changes in organizational culture   Unintended organizational outcomes (gaming, shirking, shaming, data falsification, etc.) |
|  | Population health outputs and outcomes (defined as aggregate, health and equity effects accruing defined populations) | Open answer, refers to the following outcomes:   - Utilization of specific primary care services; - Coverage of specific services or interventions (example: proportion of pregnant women receiving antenatal care; proportion of pregnant women delivering in facilities; coverage rate of specific vaccines); - Access to primary care services (example waiting times) - Health equity effects - Adverse health effects or harm - Unintended population health outcomes |
|  | Social outcomes (defined as non-health, social, economic, or cultural effects affecting defined populations) | Open answer, refers to the following outcomes:   - Community participation - Other equity effects - Other unintended social outcomes |
|  | How does this study consider equity? | - Does not address equity - Contains equity-sensitive analytical frameworks / theory of change - Uses equity-sensitive research questions - Follows equity-sensitive methodologies (sub-group analysis) - Contains equity-sensitive methodologies: additional study components to assess how and why (including mixed and qualitative methods) - Uses any other methodology that is equity sensitive that is not covered by the other options - Uses equity-informed research processes (who are the respondents, who collects data, when, where etc.) - Addresses interventions targeting specific vulnerable groups - Looks at the impact of an intervention that targets specific population groups - Measures effects on an inequality outcome |
| **Equity Data** | Dimension of equity/ Population group | Open answer, refers to the following outcomes:   - Other non-health equity effects - Adverse non-health effects or harm - Community participation - Other unintended social outcomes |
|  | Dimension of equity/ Population description | - Place of residence - Ethnicity, culture and language - Gender - Religion - Education - Socioeconomic status - Social capital - Age - Disability - Other (vulnerable group not typified by any of the above) |
|  | Equity description | Open answer – provide a description of how the study considers equity, and for which group. This is to corroborate and elaborate on the answers above. For example, describe the sub-group analysis undertaken, how the intervention targets a disadvantaged group or how the authors used an equity sensitive framework to inform their study. Please also note the page number where this information can be found. |
| **Review confidence** | Confidence in review (taken from quality appraisal from the adapted version of the SURE checklist) - (Systematic review only) | - High - Medium - Low |
|  | If high confidence, summary of findings - (Systematic review only) | Open answer |
| **Access** | Link | Open answer (if already on 3ie database, please use this link) |
|  | On 3ie database (yet)? | Yes/No |
|  |  |  |

**Data to be collected and summarized for database entries: terms of reference**

1. Existing record on database: YES/ NO (Check if there is an existing record on the database, if yes, work from this and update it, if no proceed making a new record)
2. Title:
3. Author:
4. Geographical coverage: Please list with standard regions used for the database. These are: *East Asia and Pacific (including South East Asia), South Asia, Europe and CIS, , Middle East and North Africa, Sub-Saharan Africa, Latin America and the Caribbean, North America, Commonwealth of Independent States, Developed Countries*
5. Sector: Please use list with standard sectors for the database. More than one sector category, or more sub-sectors within this category, may apply. If in doubt, err on the side of being over-inclusive, and leave a comment for the peer reviewer indicating any doubts. These are: *reproductive, maternal, newborn, child or adolescent*
6. Sub-sector: See notes at the bottom of the document for full list of sub-sectors*
7. Equity focus: this might be no, or one/ several of the following: *gender, indigenous groups, ethnic minorities, differently-abled, orphans and vulnerable children, elderly, migrant workers, refugees.* If equity considered, please state in what way.
8. Status: Completed, Protocol or Title stage (delete as appropriate)
9. Review type: effectiveness review (drawing on evidence from impact evaluations), or other review (delete as appropriate)
10. Quantitative synthesis method, if applicable: e.g.: narrative/thematic synthesis; meta-analysis etc?
11. Qualitative synthesis method, if applicable:
12. For systematic reviews rated as high-confidence only:
13. Background: Brief description of the interventions and motivation for the review. What is the problem? What is the intervention? How does it aim to have an impact on outcomes of interest?
14. Objectives: Objectives of the review – here you can quote authors verbatim, using quotation marks. But the authors stated objectives are usually a bit too long/wordy and/or any one statement does not always contain all of the objectives (this information might be at several places in the review). Rewording is almost always possible and the resulting reworded statement is usually more concise and more easily understandable, so when please reword the objectives when possible.
15. Main findings: This section should include a brief description of findings, including number of included studies, location of studies, results of synthesis (if meta-analysis was conducted, include pooled effect size and 95 percent confidence interval for relevant syntheses), conclusions, findings regarding methodology and future research.

The section should be structured as follows (including the subheadings):

One line summary: summarise the conclusions of the review in one or two sentences

Evidence base: Number and types of studies, geographical location and thematic focus

Policy relevant findings: findings related to the effectiveness of the intervention etc

Implications for further research: any mention of issues to be addressed in future research, including issues relating to study design.

1. Methodology: Inclusion criteria (including population, intervention, study design (e.g.: RCTs and Quasi-experimental studies), outcomes and contexts), outline of search (including main databases and time period of search, data collection and synthesis. Please see guidelines for more guidance on this section.
2. Applicability/external validity: Does the review discuss how generalizable the results are? What methods, if any, does the review use to assess applicability/external validity? Do the authors take any steps to improve the applicability/external validity of the findings of the review (eg: use a theory-based approach, drawing on a logic model or program theory, and/ or reporting information along the causal chain)
3. Publication Source: Author, year, Title, Publication details (if journal: title, vol, no, pp; if report series/ working paper etc.: Title of series and no if applicable, publisher location: publisher name; if book: publisher location: publisher name)

Example from JDEFF citation for journal article citation (for more refer JDEFF)

Salazar, D.J. and Alper, D.K. (2002) Reconciling environmentalism and the left: perspectives on democracy and social justice in British Columbia's environmental movement. Canadian Journal of Political Science, 35(4), pp. 527–566.

1. Downloadable link: Include link to downloadable paper – preferably open access, but if this is not available include link to pay per view.
2. Contact details of corresponding author/s:
3. Summary of quality assessment (from C3 in checklist below):
