# Supplementary material for: Evidence gap map of performance measurement and management in primary care delivery systems in low- and middle-income countries – Study protocol
Source: Gates Open Res. 2018 Nov 2;2:27. Originally published 2018 May 29. [Version 2] doi: 10.12688/gatesopenres.12826.2 (PMC6030397; doi:10.12688/gatesopenres.12826.2)
Supplement: Supplementary file 3 [file gatesopenres-2-13958-s0002.tgz › 877c6fa0-652e-4ece-8ee3-3f3108aedd91.docx]

**Supplementary file 3 – Systematic reviews critical appraisal checklist (adapted version of SURE checklist)^[[1]](#endnote-1)^**

| **Assessed by**: |
| --- |
| **Date**: |

**Section A:** *Methods used to identify, include and critically appraise studies*

| **A.1 Were the criteria used for deciding which studies to include in the review reported?**  Did the authors specify:  🞏 Types of studies  🞏 Participants/ settings/ population  🞏 Intervention(s)  🞏 Outcome(s) | 🞏 Yes  🞏 Partially  🞏 No  *Coding guide - check the answers above*  *YES: All four should be yes*  *NO: All four should be no*  *PARTIALLY: Any other* |
| --- | --- |
| *Comments (note important limitations or uncertainty)* | |
| **A.2 Was the search for evidence reasonably comprehensive?**  Were the following done:  🞏 Language bias avoided (no restriction of inclusion based on language)  🞏 No restriction of inclusion based on publication status  🞏 Relevant databases searched (Minimum criteria: All reviews should search at least one source of grey literature such as Google; for health: Medline/ Pubmed + Cochrane Library; for social sciences IDEAS + at least one database of general social science literature and one subject specific database)  🞏 Reference lists in included articles checked  🞏 Authors/experts contacted | 🞏 Yes  🞏 Partially  🞏 No  🞏 Can’t tell  *Coding guide - check the answers above:*  *YES: All five should be yes*  *PARTIALLY: Relevant databases and reference lists are both reported*  *NO: Any other* |
| *Comments (note important limitations or uncertainty)* | |
| **A.3 Does the review cover an appropriate time period?**  *Is the search period comprehensive enough that relevant literature is unlikely to be omitted?* | 🞏 Yes  🞏 Can't tell (only use if no information about time period for search)  🞏 No  🞏Unsure  *Coding guide:*  *YES: Generally, this means searching the literature at least back to 1990*  *NO: Generally, if the search does not go back to 1990*  *CAN’T TELL: No information about time period for search*  *Note: With reference to the above – there may be important reasons for adopting different dates for the search, e.g. depending on the intervention. If you think there are limitations with the timeframe adopted for the search which have not been noted and justified by the authors, you should code this item as a NO and specify your reason for doing so in the comment box below. Older reviews should not be downgraded, but the fact that the search was conducted some time ago should be noted in the quality assessment. Always report the time period for the search in the comment box.* |
| *Comments (note search period, any justification provided for the search period, or uncertainty)* | |

| **A.4 Was bias in the selection of articles avoided?**  Did the authors specify:  🞏 Independent screening of full text by at least 2 reviewers  🞏 List of included studies provided  🞏 List of excluded studies provided | 🞏 Yes  🞏 Partially  🞏 No  *Coding guide:*  *YES: All three should be yes, although reviews published in journals are unlikely to have a list of excluded studies (due to limits on word count) and the review should not be penalised for this.*  *PARTIALLY: Independent screening and list of included studies provided are both reported*  *NO: All other. If list of included studies provided, but the authors do not report whether or not the screening has been done by 2 reviewers review is downgraded to NO.* |
| --- | --- |
| *Comments (note important limitations or uncertainty):* | |
| **A.5 Did the authors use appropriate criteria to assess the quality and risk of bias in analyzing the studies that are included?^[[2]](#endnote-2)^**  🞏 The criteria used for assessing the quality/ risk of bias were reported  🞏 A table or summary of the assessment of each included study for each criterion was reported  🞏 Sensible criteria were used that focus on the quality/ risk of bias (and not other qualities of the studies, such as precision or applicability/external validity). “Sensible” is defined as a recognized quality appraisal tool/ checklist, or similar tool which assesses bias in included studies. Please see footnotes for details of the main types of bias such a tool should assess. | 🞏 Yes  🞏 Partially  🞏 No  *Coding guide:*  *YES: All three should be yes*  *PARTIALLY: The first and third criteria should be reported. If the authors report the criteria for assessing risk of bias and report a summary of this assessment for each criterion, but the criteria may be only partially sensible (e.g. do not address all possible risks of bias, but do address some), we downgrade to PARTIALLY.*  *NO: Any other* |
| *Comments (note important limitations or uncertainty)* | |
| **A.6 Overall – how much confidence do you have in the methods used to identify, include and critically appraise studies?**  *Summary assessment score A relates to the 5 questions above.*  *High confidence applicable when the answers to the questions in section A are all assessed as ‘yes’*  *Low confidence applicable when any of the following are assessed as ‘NO’ above: not reporting explicit selection criteria (A1), not conducting reasonably comprehensive search (A2), not avoiding bias in selection of articles (A4 , not assessing the risk of bias in included studies (A5)*  *Medium confidence applicable for any other – i.e. section A3 is assessed as ‘NO’ or can’t tell and remaining sections are assessed as ‘partially’ or ‘can’t tell’* | 🞏 **Low confidence** (limitations are important enough that the results of the review are not reliable)  🞏 **Medium confidence** (limitations are important enough that it would be worthwhile to search for another systematic review and to interpret the results of this review cautiously, if a better review cannot be found)  🞏 **High confidence** (only minor limitations) |
| *Comments (note important limitations).* | |

**Section B:** *Methods used to analyze the findings*

| **B.1 Were the characteristics and results of the included studies reliably reported?**  Was there:  🞏 Independent data extraction by at least 2 reviewers  🞏 A table or summary of the characteristics of the participants, interventions and outcomes for the included studies  🞏 A table or summary of the results of all the included studies | 🞏 Yes  🞏 No  🞏 Partially  🞏 Not applicable (e.g. no included studies)  *Coding guide:*  *YES: All three should be yes*  *PARTIALLY: Criteria one and three are yes, but some information is lacking on second criteria.*  *No: None of these are reported. If the review does not report whether data was independently extracted by 2 reviewers (possibly a reporting error), we downgrade to NO.*  *NOT APPLICABLE: if no studies/no data* |
| --- | --- |
| *Comments (note important limitations or uncertainty)* | |
| **B.2 Are the methods used by the review authors to analyze the findings of the included studies clear, including methods for calculating effect sizes if applicable?** | 🞏 Yes  🞏 Partially  🞏 No  🞏 Not applicable (e.g. no studies or no data)  *Coding guide:*  *YES: Methods used clearly reported. If it is clear that the authors use narrative synthesis, they don't need to say this explicitly.*  *PARTIALLY: Some reporting on methods but lack of clarity*  *NO: Nothing reported on methods*  *NOT APPLICABLE: if no studies/no data* |
| *Comments (note important limitations or uncertainty)* | |
| **B.3 Did the review describe the extent of heterogeneity?**  🞏 Did the review ensure that included studies were similar enough that it made sense to combine them, sensibly divide the included studies into homogeneous groups, or sensibly conclude that it did not make sense to combine or group the included studies?  🞏 Did the review discuss the extent to which there were important differences in the results of the included studies?  🞏 If a meta-analysis was done, was the I^2^, chi square test for heterogeneity or other appropriate statistic reported? If no statistical test was reported, is a qualitative justification made for the use of random effects? | 🞏 Yes  🞏 Partially  🞏 No  🞏 Not applicable (e.g. no studies or no data)  *Coding guide:*  *YES: First two should be yes, and third category should be yes if applicable should be yes*  *PARTIALLY: The first category is yes*  *NO: Any other*  *NOT APPLICABLE: if no studies/no data* |
| *Comments (note important limitations or uncertainty)* | |

| **B.4 Were the findings of the relevant studies combined (or not combined) appropriately relative to the primary question the review addresses and the available data?**  How was the data analysis done?  🞏 Descriptive only  🞏 Vote counting based on direction of effect  🞏 Vote counting based on statistical significance  🞏 Description of range of effect sizes  🞏 Meta-analysis  🞏 Meta-regression  🞏 Other: specify  🞏 Not applicable (e.g. no studies or no data)  How were the studies weighted in the analysis?  🞏 Equal weights (this is what is done when vote counting is used)  🞏 By quality or study design (this is rarely done)  🞏 Inverse variance (this is what is typically done in a meta-analysis)  🞏 Number of participants (sample size)  🞏 Other: specify  🞏 Not clear  🞏 Not applicable (e.g. no studies or no data)  Did the review address unit of analysis errors?  🞏 Yes - took clustering into account in the analysis (e.g. used intra-cluster correlation coefficient)  🞏 No, but acknowledged problem of unit of analysis errors  🞏 No mention of issue  🞏 Not applicable - no clustered trials or studies included | 🞏 Yes  🞏 Partially  🞏 No  🞏 Not applicable (e.g. no studies or no data)  🞏 Can’t tell  *Coding guide:*  *YES: If appropriate table, graph or meta-analysis AND appropriate weights AND unit of analysis errors addressed (if appropriate).*  *PARTIALLY: If appropriate table, graph or meta-analysis AND appropriate weights AND unit of analysis errors not addressed (and should have been).*  *NO: If narrative OR vote counting (where quantitative analyses would have been possible) OR inappropriate reporting of table, graph or meta-analyses.*  *NOT APPLICABLE: if no studies/no data*  *CAN’T TELL: if unsure (note reasons in comments below)* |
| --- | --- |
| *Comments (note important limitations or uncertainty)* | |
| **B. 5 Does the review report evidence appropriately?**  🞏 The review makes clear which evidence is subject to low risk of bias in assessing causality (attribution of outcomes to intervention), and which is likely to be biased, and does so appropriately  🞏 Where studies of differing risk of bias are included, results are reported and analyzed separately by risk of bias status | 🞏 Yes  🞏 No  🞏 Partially  🞏 Not applicable  *Coding guide:*  *YES: Both criteria should be fulfilled (where applicable)*  *NO: Criteria not fulfilled*  *PARTIALLY: Only one criteria fulfilled, or when there is limited reporting of quality appraisal (the latter applies only when inclusion criteria for study design are appropriate)*  *NOT APPLICABLE: No included studies*  *Note on reporting evidence and risk of bias: For reviews of effects of ‘large n’ interventions, experimental and quasi-experimental designs should be included (if available). For reviews of effects of ‘small n’ interventions, designs appropriate to attribute changes to the intervention should be included (e.g. pre-post with assessment of confounders)* |
| *Please specify included study designs and any other comments (note important limitations or uncertainty):* | |
| **B.6 Did the review examine the extent to which specific factors might explain differences in the results of the included studies?**  🞏 Were factors that the review authors considered as likely explanatory factors clearly described?  🞏 Was a sensible method used to explore the extent to which key factors explained heterogeneity?  🞏 Descriptive/textual  🞏 Graphical  🞏 Meta-analysis by sub-groups  🞏 Meta-regression  🞏 Other | 🞏 Yes  🞏 Partially  🞏 No  🞏 Not applicable  *Coding guide:*  *YES: Explanatory factors clearly described and appropriate methods used to explore heterogeneity*  *PARTIALLY: Explanatory factors described but for meta-analyses, sub-group analysis or meta-regression not reported (when they should have been)*  *NO: No description or analysis of likely explanatory factors*  *NOT APPLICABLE: e.g. too few studies, no important differences in the results of the included studies, or the included studies were so dissimilar that it would not make sense to explore heterogeneity of the results* |
| *Comments (note important limitations or uncertainty)* | |
| **B.7 Overall - how much confidence do you have in the methods used to analyze the findings relative to the primary question addressed in the review?**  *Summary assessment score B relates to the 5 questions in this section, regarding the analysis.*  *High confidence applicable when all the answers to the questions in section B are assessed as ‘yes’.*  *Low confidence applicable when any of the following are assessed as ‘NO’ above: critical characteristics of the included studies not reported (B1), not describing the extent of heterogeneity (B3), combining results inappropriately (B4), reporting evidence inappropriately (B5).*  *Medium confidence applicable for any other: i.e. the “Partial” option is used for any of the 6 preceding questions or questions and/or B.2 and/ or B.6 are assessed as ‘no’.* | 🞏 **Low confidence** (limitations are important enough that the results of the review are not reliable)  🞏 **Medium confidence** (limitations are important enough that it would be worthwhile to search for another systematic review and to interpret the results of this review cautiously, if a better review cannot be found)  🞏 **High confidence** (only minor limitations) |
| *Use comments to specify if relevant, to flag uncertainty or need for discussion* | |

**Section C: Overall assessment of the reliability of the review**

| **C.1 Are there any other aspects of the review not mentioned before which lead you to question the results?** | 🞏 Additional methodological concerns – only one person reviewing  🞏 Robustness  🞏 Interpretation  🞏 Conflicts of interest (of the review authors or for included studies)  🞏 Other  🞏 No other quality issues identified |
| --- | --- |
| **C.2 Are there any mitigating factors which should be taken into account in determining the reviews reliability?** | 🞏 Limitations acknowledged  🞏 No strong policy conclusions drawn (including in abstract/ summary)  🞏 Any other factors |
| *Use comments to specify if relevant, to flag uncertainty or need for discussion* | |
| C.3 **Based on the above assessments of the methods how would you rate the reliability of the review?**  🞏 **Low confidence in conclusions about effects**:  The systematic review has the following major limitations...  🞏 **Medium confidence in conclusions about effects**:  The systematic review has the following limitations...  🞏 **High confidence in conclusions about effects:**  If applicable: The review has the following minor limitations...  *Coding guide:*  **High confidence in conclusions about effects**: high confidence noted overall for sections A and B, unless moderated by answer to C1.  **Medium confidence in conclusions about effects**: medium confidence noted overall for sections A or B, unless moderated by answer to C1 or C2.  **Low confidence in conclusions about effects**: low confidence noted overall for sections A or B, unless moderated by answer to C1 or C2.  Limitations should be summarised above, based on what was noted in Sections A, B and C. | |

**NOTES**

1. Adapted from Supporting the Use of Research Evidence (SURE) Collaboration. SURE checklist for making judgements about how much confidence to place in a systematic review. In: SURE guides for preparing and using policy briefs. [www.evipnet.org/sure](http://www.evipnet.org/sure) [↑](#endnote-ref-1)
2. **Risk of bias** is the extent to which bias may be responsible for the findings of a study.

   **Bias** is a systematic error or deviation from the truth in results or inferences. In studies of the effects of social, economic and health care interventions, the main types of bias arise from systematic differences in the groups that are compared (selection bias), the intervention that is provided, or exposure to other factors apart from the intervention of interest (performance bias/contamination), withdrawals or exclusions of people entered into a study (attrition bias) or how outcomes are assessed (detection bias) and reported (reporting bias). Reviews of social science studies may be particularly affected by reporting bias, where a biased subset of all the relevant data and analyses is presented.

   Assessments of the risk of bias are sometimes also referred to as assessments of the **validity** or **quality** of a study.

   **Validity** is the extent to which a result (of a measurement or study) is likely to be true.

   **Quality** is a vague notion of the strength or validity of a study, often indicating the extent of control over bias.

   ***Question 6: Full list of sub-sectors by sector**

   Agriculture and Rural Development

   *Agricultural Reform; Agricultural Credit; Agricultural Extension; Agro-Industry & Marketing; Weather Insurance; Fisheries & Aquaculture; Forestry; Irrigation & Drainage; Livestock; Agricultural Research; Rural Land Reform; Rural Livelihoods; Rural Roads*

   Economic Policy

   *Macro/ Non-Trade; Trade; Tax Reform*

   Education

   *Distance Education/ Education Technology; Educational Inputs; Girl's Education; Non-formal Education; Pre-Primary and Primary Education; Public/ Private Sector Education; Secondary Education; Student Loan; System Reform & Capacity Building; Tertiary Education; Vocational/ Technical Education & Training*

   Energy

   *Distribution & Transmission; Hydro; Thermal; Other Power & Energy Conversion; Rural Electrification*

   Environment and Disaster Management

   *Biodiversity; Climate Change; Environmental Institutions; Natural Resource Management; Pollution Control/ Waste Management; Resettlement; Disaster Relief*

   Finance

   *Capital Markets Development; Banking Systems; Consumer Credit; Financial Sector Reform; Microfinance;*

   Health Nutrition and Population

   *Child Nutrition; Nutrition; Health Sector Reform; Health Financing, Insurance and User Fees; Health Services; HIV/AIDS; Hospitals- Secondary & Tertiary; Mortality; Preventive Health and Health Behavior; Primary Health- including reproductive health; Sexual Behavior; Specific Diseases- including Malaria TB; Specific Diseases-diarrhoea.*

   Information and Communication Technology

   *Telecommunications Mass Media Technological Innovation Technology Development Funds*

   Multisector

   *Community Action Program; Community Driven Development; Concessions; Conditional Cash Transfers; Conflict Prevention and Post-Conflict Reconstruction; Social Funds; Early Childhood Development Programs; Multisector*

   Private Sector Development

   *Business Environment; Private Infrastructure; Small Scale Enterprise*

   Public Sector Management

   *Anti-corruption/ Governance; Civil Service Reform; Decentralization; Institutional Development; Judicial Reform; Public Financial Management*

   Social Protection

   *Labor markets & Employment; Pensions & Social Insurance; Savings and Remittances; Social Assistance; Social Protection Reform*

   Transportation

   *Highways; Ports and Waterways Railways*

   Urban Development

   *Urban housing; Urban land reform; Urban transport; Slum upgradation Programmes; Urban development and management*

   Water and Sanitation

   *Water Supply and Sanitation Reform; Urban Water and Sanitation; Rural Water and Sanitation* [↑](#endnote-ref-2)
