# Supplementary material for: Evidence gap map of performance measurement and management in primary care delivery systems in low- and middle-income countries – Study protocol
Source: Gates Open Res. 2018 Nov 2;2:27. Originally published 2018 May 29. [Version 2] doi: 10.12688/gatesopenres.12826.2 (PMC6030397; doi:10.12688/gatesopenres.12826.2)
Supplement: Peer review history

# Peer review: Reviewer response for version 2

**DOI:** 10.21956/gatesopenres.13958.r26734

**Published:** 2018-11-06

**Authors:** Eliudi Eliakimu

**Affiliations:** 1 Health Services Inspectorate and Quality Assurance Section under the Health Quality Assurance Division, Ministry of Health, Community Development, Gender, Elderly and Children, Dodoma, Tanzania

I approve the paper. The authors have addressed well the previous comments. However, there is a very minor error that needs correction on page 6 of 19 of version 2 of their paper ^1^. The authors have noted in the paragraph under the sub-heading “specific intervention and outcomes of interest” that “the study will focus on **two”** categories while they have listed **three **general categories from the EPOC taxonomy ^2^. Therefore, I suggest to correct it so that it will read as follows – “the study will focus on three: …….” Also, on page 9 of 19 under the sub-title “types of included study designs”, in the first bullet, the reference “Snilstveit *et al*, 2018” - it is not clear if it is forthcoming or in progress, since it is missing in the reference list. I suggest that the authors need to clarify it more and consider adding it to the reference list as they will see appropriate.

I have read this submission. I believe that I have an appropriate level of expertise to confirm that it is of an acceptable scientific standard.

## References

[1] MunarWSnilstveitBStevensonJBiswasNEyersJButeraGBaffourTArandaL: Evidence gap map of performance measurement and management in primary care delivery systems in low- and middle-income countries – Study protocol. *Gates Open Research*.2018;2: 10.12688/gatesopenres.12826.2 10.12688/gatesopenres.12826.2 29984360PMC6030397

[2] Effective Practice and Organisation of Care (EPOC). *EPOC Taxonomy*.2015; Reference source

# Response

**Published:** 2018-11-06

**Authors:** Wolfgang Munar

Much appreciated. Thanks a lot!

 Wolfgang

# Peer review: Reviewer response for version 1

**DOI:** 10.21956/gatesopenres.13899.r26534

**Published:** 2018-07-02

**Authors:** Eliudi Eliakimu

**Affiliations:** 1 Health Services Inspectorate and Quality Assurance Section under the Health Quality Assurance Division, Ministry of Health, Community Development, Gender, Elderly and Children, Dodoma, Tanzania

As all countries of the World work towards attainment of the Sustainable Development Goals (SDGs) including SDG-3: “ *Ensure healthy lives and promote well-being for all at all ages*”, the need for a stronger Primary Health Care (PHC) System is evident. The need for a well-functioning PHC systems particularly in Low and Middle-Income Countries (LMICs) is critical given the fact that stronger PHC system in LMICs is essential for achieving the Universal Health Coverage (UHC) target ^1^. The purpose of this referee report is to review the article on “ *Evidence Gap Map of Performance Measurement and Management in Primary Health Care Delivery Systems in Low and Middle-Income Countries…..”*  by Munar et al ^2^ to examine whether it is scientifically sound. The referee report examine the article based on the following parts as they appear in the paper: background; interventions and outcomes of interest; how performance measurement and management may work in primary care systems; study objectives; methods; discussion; dissemination of findings; supplementary materials; and references.

***Background*** – The authors have presented well the background information and indeed they have done good job by bringing in an interdisciplinary perspective in the performance measurement and management in PHC in LMICs. Their generic framework presented in figure 1 is comprehensive enough and it clearly shows the interplay between the performance measurement and management steps and the context in health sector which encompass both institutional and organizational contexts as well as the context outside the health sector. This brings a robust perspective on what constitutes performance of PHC system in LMICs.

***Interventions and outcomes of interest*** – The authors have clearly defined the key concepts involved and description of the interventions and outcomes of interest. This part is well presented, however the authors need to work on the following minor issues to improve this part. First, table 1 in page 5 of 14 contains a total of 12 interventions (i. Clinical practice guidelines; ii. reminders; iii. in-service training; iv. continuous education; v. supervision; vi. clinical incident reporting; vii. local opinion leaders; viii. continuous quality improvement [including lean]; ix. performance based financing; x. public release of performance information; xi. social accountability; and xii. audit and feedback), which are spread in the three categories of interventions, but the authors have made an error by indicating that in total there are 11 interventions. Secondly, they need to correct spelling error for the word “accountability” in right column middle row where it refers to social accountability. Also, on the same page 5 of 14, in the paragraph with subheading “accountability arrangements”, the authors need to check the spellings for “intervention” in the fourth line in that paragraph since it is not clear whether they wanted to say “such intervention” or “such interventions”. The outcomes included in Table 2 on page 6 of 14 in the column named as “ *provider and managerial outputs and outcomes*” are appropriate, however, I suggest to the authors to consider adding provider availability at work (i.e., looking at absenteeism of health workers in health facilities). This is an important management element in health facilities in LMICs. For example, in Tanzania, absenteeism has been documented to be 14% and in other LMICs it has been documented to be in a range of 14%-–60% ^3^^,^^4^. Some countries are trying to address this challenge, for example, as a way of improving performance management in PHC facilities in Tanzania through an initiative referred to as Big Results Now (BRN), the delivery bureau through stakeholders consultations in a setting contextualized as “Laboratory” they identified use of timesheet as one of measures to address absenteeism. Therefore, I strongly recommend to the authors to consider this as part of the managerial outputs and outcomes. Also, on page 6 of 14 in the paragraph starting with “The main categories included in this EGM…….” The fourth category “4) population-level……… equity effects accruing defined population…,” I suggest to add the word “in” between accruing and defined.

***How performance measurement and management may work in primary care systems*** – This part is well presented and figure 2 that depicts the authors’ framework for performance measurement and management in PHC systems is very comprehensive with clear linkages between its components.

***Study objectives*** – The objectives of the study are well presented and succinct. I am convinced that upon realization of the objectives, the outputs of the study will synergize very well with the work done so far (as well as ongoing work) in the Primary Health Care Performance Initiative (PHCPI) ^5^.

***Methods*** – The authors have presented a detailed methodology which is well organized narrating the inclusion and exclusion criteria; the types of study designs that are included; search strategy; screening procedure; and statistical analysis and EGM visualization. To the best of my knowledge this part is correct. There is a very minor correction on page 9 of 14 under the subheading *“procedures for screening and data extraction*”, specifically in the second paragraph (fifth line), they need to change the word “includes” to read as “included”, hence the sentence will read: ……..” *most likely to be included based on*….”

   

***Discussion ***– It is brief and clear that orients a reader on what is an EGM and its value in decision making which is in line with the existing literature. However, I suggest that the authors need to add a bit of information on how to read the EGM so that readers (including decision makers) who are new to the concept can easily follow and understand the EGM when it is released and disseminated.

   

***Dissemination of findings*** – Excellent explanations on how to disseminate the findings including the use of social media. However, the authors need to correct the year for the 5 ^th^ Global Health System Symposium. I hope that they mean “ *the 5 ^th^ Global Health System Symposium in October 2018 and not 2017*” as indicated in the paper on page 11 of 14.

***Supplementary materials*** – The supplementary materials are well presented and comprehensive. The search strategy (File 1) is comprehensive. The coding tools (File 2) is also comprehensive and well presented, however, on page 2 and 6 in the middle column under the heading “ *population health outputs and outcomes (defined as………..effects accruing defined populations)*”, there is a need to add “in” between accruing and defined. File 3 contains a very comprehensive checklist.

***References*** – The reference list is correctly presented.

I have read this submission. I believe that I have an appropriate level of expertise to confirm that it is of an acceptable scientific standard.

## References

[1] van WeelCKiddMR: Why strengthening primary health care is essential to achieving universal health coverage. *CMAJ*.2018;190(15) : 10.1503/cmaj.170784 E463-E466 10.1503/cmaj.170784 29661815PMC5903888

[2] MunarWSnilstveitBStevensonJBiswasNEyersJButeraGBaffourTArandaL: Evidence gap map of performance measurement and management in primary care delivery systems in low- and middle-income countries – Study protocol. *Gates Open Research*.2018;2: 10.12688/gatesopenres.12826.1 10.12688/gatesopenres.12826.1 29984360PMC6030397

[3] DuganiSVeillardJEvansTG: Quality primary health care will drive the realization of universal health coverage. *CMAJ*.2018;190(15) : 10.1503/cmaj.180186 E453-E454 10.1503/cmaj.180186 29661813PMC5903886

[4] DasJWoskieLRajbhandariRAbbasiKJhaA: Rethinking assumptions about delivery of healthcare: implications for universal health coverage. *BMJ*.2018;361:k1716 2978487010.1136/bmj.k1716PMC5961312

[5] VeillardJCowlingKBittonARatcliffeHKimballMBarkleySMercereauLWongETaylorCHirschhornLRWangH: Better Measurement for Performance Improvement in Low- and Middle-Income Countries: The Primary Health Care Performance Initiative (PHCPI) Experience of Conceptual Framework Development and Indicator Selection. *Milbank Q*.95(4) : 10.1111/1468-0009.12301 836-883 10.1111/1468-0009.12301 29226448PMC5723717

# Response

**Published:** 2018-07-02

**Authors:** Wolfgang Munar

Dear Prof. Eliakimu: On behalf of the authors, we all appreciate your detailed comments very much. We will make sure to edit and update the paper to reflect them. The well-founded suggestion regarding absenteeism is quite relevant. The team will discuss and make a decision about its inclusion in the evidence gap map.

 Wolfgang Munar

# Peer review: Reviewer response for version 1

**DOI:** 10.21956/gatesopenres.13899.r26476

**Published:** 2018-06-25

**Authors:** Oscar Bernal-Acevedo

**Affiliations:** 1 Facultad de Medicina, Universidad de Los Andes, Bogotá, Colombia

The research is pertinent due to the lack of evidence about primary health care performance in low- and middle-income countries. The objective is clear however the goal of this specific article is to describe the gap map methodology as a first step to develop the gap map.

 I recommended to differentiate between low- and middle-income countries because they have very different context and capacity to produce and publish evidence on primary health care.

 The use of the International Initiative for Impact Evaluation allows the researchers to compare the results with other studies and to have a standard methodology.

 The performance management system included implementation strategies and financial arrangements. However, the methodology excluded demand-side interventions (financial).

 The performance framework in primary care delivery systems is already an important product of this research including evaluation cycle and context. However, the link between the two is not clear, the two directions arrow is not enough to explain the interaction between the context and the outcomes.

 The framework is an excellent exercise combining interventions, outputs and outcomes. But, the context is not present in this framework. Some indicators need to be more precise, for example the “number of antenatal visits” (% of pregnant women with more than 2,3 or 4 antenatal visits?)

 The use of grey literature is an important aspect of this project because most of the evaluations on primary health care intervention has not been published in peer journals. I recommended to add web pages from Ministries of Health and Bireme because they have a data base on grey literature. The research will review only articles in English, I recommend to included French and Spanish to increase the countries and articles from Latin America and some African countries.

 The discussion not included the limitations of this study, related for example to the limited quality of data in low- and middle-income countries.

 They mention that the “Findings will be presented at the 5th Global Health System Symposium in October 2017” but the article is not published until now.

 The bibliography is extensive and updated and show a big effort in terms of analysis of existing evidence.

I have read this submission. I believe that I have an appropriate level of expertise to confirm that it is of an acceptable scientific standard.

# Response

**Published:** 2018-07-02

**Authors:** Wolfgang Munar

Dear Prof. Bernal-Acevedo- thanks a lot for your comments. They are all relevant and applicable. Your comment about the generic and possibly inappropriate aggregation of low- and middle-income countries is one that we expect to address in the findings as it may well require definitions that differentiate institutional and organizational contexts in addition to, the conventional income-based definitions used in the "LMIC" construct.

 Wolfgang Munar on behalf of the authors
